# MRI-based prediction of the need for wide resection margins in patients with single hepatocellular carcinoma

**DOI:** 10.1007/s00330-024-11043-5

**Published:** 2024-09-05

**Authors:** Yanshu Wang, Yali Qu, Chongtu Yang, Yuanan Wu, Hong Wei, Yun Qin, Jie Yang, Tianying Zheng, Jie Chen, Roberto Cannella, Federica Vernuccio, Maxime Ronot, Weixia Chen, Bin Song, Hanyu Jiang

**Affiliations:** 1 Department of Radiology, West China Hospital, Sichuan University, Chengdu, China; 2 Department of Radiology, Functional and Molecular Imaging Key Laboratory of Sichuan Province, West China Hospital, Sichuan University, Chengdu, China; 3 Big Data Research Center, University of Electronic Science and Technology of China, Chengdu, China; 4 Department of Medical Ultrasound, West China Hospital, Sichuan University, Chengdu, China; 5 Department of Biomedicine, Neuroscience and Advanced Diagnostics, University of Palermo, Palermo, Italy; 6 Université Paris Cité, UMR 1149, CRI, Paris & Service de Radiologie, Hôpital Beaujon, APHP.Nord, Clichy, France; 7 Department of Radiology, Sanya People’s Hospital, Sanya, China

**Keywords:** Hepatocellular carcinoma, Magnetic resonance imaging, Wide resection margin, Early recurrence

## Abstract

**Objectives:**

To develop an MRI-based score that enables individualized predictions of the survival benefit of wide over narrow resection margins.

**Materials and methods:**

This single-center retrospective study (December 2011 to May 2022) included consecutive patients who underwent curative-intent resection for single Barcelona Clinic Liver Cancer (BCLC) 0/A HCC and preoperative contrast-enhanced MRI. In patients with narrow resection margins, preoperative demographic, laboratory, and MRI variables independently associated with early recurrence-free survival (RFS) were identified using Cox regression analyses, which were employed to develop a predictive score (named “MARGIN”). Survival outcomes were compared between wide and narrow resection margins in a propensity-score matched cohort for the score-stratified low- and high-risk groups, respectively.

**Results:**

Four hundred nineteen patients (median age, 54 years; 361 men) were included, 282 (67.3%) undergoing narrow resection margins. In patients with narrow resection margins, age, alpha-fetoprotein (AFP) > 400 ng/mL, protein induced by vitamin K absence or antagonist-II (PIVKA-II) > 200 mAU/mL, radiological involvement of liver capsule, and infiltrative appearance were associated with early RFS (*p* values, 0.002–0.04) and formed the MARGIN score with a testing dataset *C*-index of 0.75 (95% CI: 0.65–0.84). In the matched cohort, wide resection margin was associated with improved early RFS rate for the high-risk group (*MARGIN* score ≥ − 1.3; 71.1% vs 41.0%; *p* = 0.02), but not for the low-risk group (*MARGIN* score < − 1.3; 79.7% vs 76.1%; *p* = 0.36).

**Conclusion:**

In patients with single BCLC 0/A HCC, the MARGIN score may serve as promising decision-making to indicate the need for wide resection margins.

**Clinical relevance statement:**

The MARGIN score has the potential to identify patients who would benefit more from wide resection margins than narrow resection margins, improving the postoperative survival of patients with single BCLC 0/A hepatocellular carcinoma (HCC).

**Key Points:**

- *Age, AFP, PIVKA-II, radiological involvement of liver capsule, and infiltrative appearance were associated with early RFS and formed the MARGIN score*.
- *The MARGIN score achieved a testing dataset C-index of 0.75*.
- *Wide resection margins were associated with improved early RFS for the high-risk group, but not for the low-risk group*.

**Graphical Abstract:**

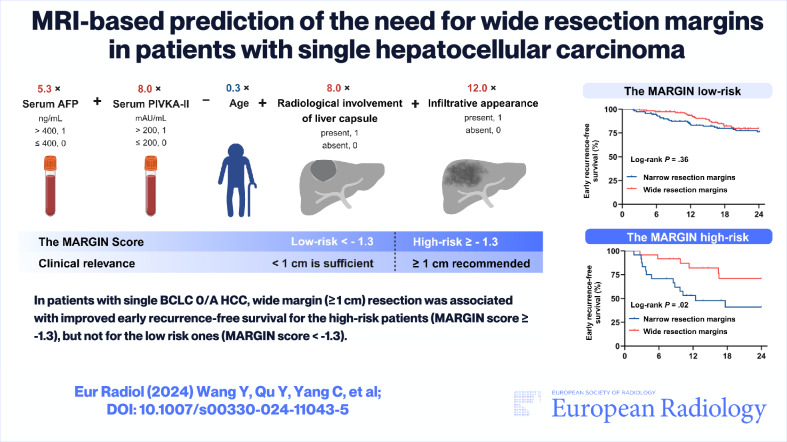

## Introduction

Surgical resection constitutes a major curative-intent treatment option for resectable hepatocellular carcinoma (HCC) [1]. One of the main challenges in the surgical planning of HCC is to determine the appropriate resection margin to ensure complete tumor removal and low risk of recurrence while preserving as much liver function as possible [1].

Since peritumoral liver parenchyma could contain satellite nodules and microvascular invasion (MVI) that might be radiologically undetectable [2], a resection margin width ≥ 1 cm is recommended to reduce postoperative early recurrence [3–5]. However, conflicting results have been reported regarding the optimal margin width [6, 7], and several studies showed limited survival benefits of wide resection margins (≥ 1 cm) compared with narrow resection margins (< 1 cm) [8, 9]. A plausible explanation for the inconsistencies in optimal margin width is that HCC is a biologically heterogeneous malignancy with variegated prognostic benefits from wide resection margins. Furthermore, there are several intrinsic limitations of wide resection margins. For instance, it may carry an increased risk for severe perioperative complications, such as life-threatening bleeding and liver failure [4, 10], and the feasibility of wide resection margins could be limited by tumor size, location, proximity to large vessels, and functional liver reserve [11, 12]. Given that wide resection margins may be potentially ineffective and risky for some patients, narrow resection margins are conducted for a large proportion of patients in clinical practice [12]. Thus, to guarantee acceptable oncological outcomes, it is crucial to identify patients for whom wide resection margins are more effective than narrow resection margins.

Previous research has reported that small tumor size (< 5 cm) [13], elevated alpha-fetoprotein (AFP, > 100 ng/mL) [14], [^18^F]FDG positive lesions [15], positive circulating tumor cell status [16], and poorly demarcated gross types [17] as single indicators favoring the selection of wide resection margin. However, as a biologically heterogeneous malignancy, the prognosis of HCC has been found to vary substantially within any category defined based on a single indicator (e.g., the post-resection survival of HCC < 5 cm varied depending on AFP levels [18]). Therefore, relying on a single indicator to estimate the survival benefit of wide resection margins in heterogeneous patient cohorts is likely inaccurate. Fortunately, imaging techniques, particularly magnetic resonance imaging (MRI), can display the whole landscape of HCC, potentially providing insights into its heterogeneity in a non-invasive manner. By considering multiple MRI features simultaneously, several MRI-based models have shown promising results for stratifying the risk of recurrence or survival and the potential to identify optimal candidates for HCC treatments [19, 20]. However, there are few studies using the MRI-based model to identify patients who would not achieve adequate oncological radicality with narrow resection margins and may be at lower risk of early recurrence with wide resection margins.

Therefore, the aim of this study was to develop an MRI-based score to support the selection between wide- and narrow resection margins, based on the prediction of postoperative early recurrence-free survival (RFS ≤ 24 months) in patients receiving narrow resection margins for a single Barcelona clinic liver cancer (BCLC) 0 or A HCC.

## Methods and materials

This single-center retrospective study was approved by the Ethics Committees of West China Hospital, Sichuan University, and the requirement to obtain written informed consent was waived.

### Patient selection

**Fig. 1:**
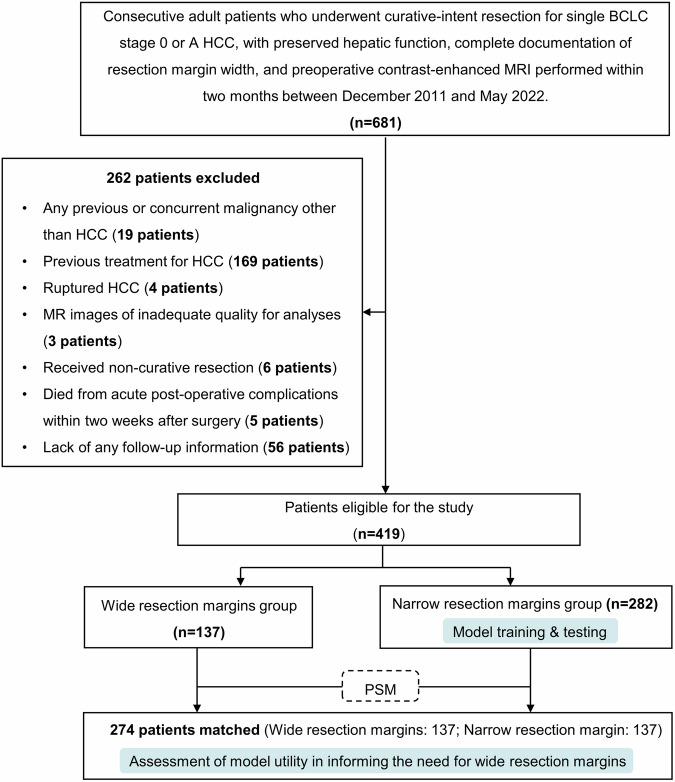
Study flowchart. HCC, hepatocellular carcinoma; PSM, propensity score-matching

Figure 1 portrays the subjects’ inclusion and exclusion flowchart, in accordance with the standards for reporting of diagnostic accuracy initiative [21].

From December 2011 to May 2022, consecutive patients who fulfilled the following inclusion criteria were identified: (a) age ≥ 18 years; (b) pathologically diagnosed single BCLC 0 or A HCC; (c) underwent curative-intent liver resection; (d) preserved hepatic function (i.e., Child-Pugh class A5 through B7); (e) complete documentation of resection margin width, and (f) underwent preoperative contrast-enhanced MRI within two months prior to surgery. The exclusion criteria were: (a) any previous or concurrent malignancy other than HCC; (b) previous treatment for HCC; (c) ruptured HCC; (d) MR images of inadequate quality for analyses; (e) received non-curative resection [22]; (f) died from acute post-operative complications within two weeks after surgery; or (g) lack of any follow-up information.

This work included 339 previously reported patients [23], but with distinct study purposes, design, and endpoints.

### Demographic, laboratory, surgical, and histologic data

Demographic, laboratory, surgical, and histopathological data were collected from electronic patient records. A wide- or a narrow-resection margin was defined as the pathological shortest distance from the edge of the tumor to the plane of liver resection being ≥ 1 cm or < 1 cm, as previously described [4]. Further details can be found in the Supplementary Material 1.

### Image acquisition and analysis

Contrast-enhanced MRI examinations using either extracellular or hepatobiliary contrast agents (HBA) were performed with various 1.5-T or 3.0-T scanners. Image acquisition protocols are detailed in Supplementary Material 2 and Supplementary Table 1.

All MRI images were evaluated by three independent fellowship-trained abdominal radiologists (with 7 years, 10 years, and 3 years of experience in liver MRI, respectively) who were aware of the HCC diagnosis but blinded to all remaining clinical, histopathological, and follow-up information. A total of 51 MRI features were evaluated, including (a) tumor location, (b) imaging features related to tumor aggressiveness (including all liver imaging reporting and data system v2018 features [24] and other previously reported prognostic features of the tumor [25]), and (c) features related to the underlying liver condition. Detailed MRI features and their definitions are shown in Supplementary Table 2. Further information on resolving inter-rater disagreements is described in Supplementary Material 2.

### Follow-up and outcome measures

After surgery, patients were followed up at one month, every three months for the first two years, and every six months thereafter until death or May 1, 2022. Serum AFP, liver biochemistry, and abdominal ultrasound or contrast-enhanced CT/MRI were included for follow-up examinations. Bone scans, chest CT, head CT/MRI, and biopsies were performed when clinically indicated.

The early RFS rate (i.e., the proportion of patients alive and recurrent-free at 24 months) was the primary endpoint for survival analyses as it has been predominantly associated with tumor aggressiveness and resection radicality [26].

### Statistical analysis

The statistical analyses are detailed in Supplemental Material 3. Briefly, based on the assumption that achieving insufficient oncologic outcomes with narrow resection margins would benefit more from wide resection margins, an MRI-based model (i.e., MARGIN) for predicting postoperative early RFS in patients with narrow resection margins was developed, which served as the basis for predicting the need for wide resection margins.

#### Score development and validation

To ensure effective multivariable regression analyses, the sample size was estimated for at least five outcome events per variable [27]. Eligible patients with narrow resection margins were randomly divided into training and testing datasets at a ratio of 7:3. In the training dataset, predictive preoperative demographic, laboratory, and MRI variables were identified using univariable and multivariable Cox regression analyses with backward step-wise selection and five-fold cross-validation (cutoffs detailed in Supplemental Table 3). Inter-variable collinearity was calculated using Spearman’s correlation analysis. A scoring system was formulated based on the statistically significant variables from the multivariable Cox analysis weighted by their respective β coefficients, with the largest β coefficient scaled as 12 points and the remaining proportionally rounded to the nearest integer to improve clinical utility. Model discrimination was evaluated using the concordance index (*C*-index) and time-dependent area under the curve (AUC). To divide the patients into high- and low-risk groups for early recurrence, the optimal threshold value of the score was determined at the 75th percentile based on the training dataset.

#### Score utility in informing the need for wide resection margins

Based on pretreatment factors that may have influenced survival and resection margin selection (i.e., cirrhosis [28], ALBI score [9], tumor size [9], and tumor location [29]), a 1:1 optimal propensity score matching was performed between patients who received narrow and wide resection margins. Survival outcomes were estimated with the Kaplan–Meier method and compared using the log-rank test. In MARGIN-predicted high-risk patients, the prognostic value of margin width was evaluated by multivariable analyses in conjunction with other previously published prognostic variables. Furthermore, details of incremental prognostic values of the MARGIN score to the preoperative Early Recurrence After Surgery for Liver Tumor (ERASL-pre) score [26] are described in the Supplementary Material.

R (version 4.2.2; R Foundation for Statistical Computing) was used for statistical analysis. A two-sided *p* < 0.05 was considered statistically significant.

## Results

### Patients

This study included 419 patients (median age, 54 years; 361 men), 282 (67.3%) with narrow resection margins (Table 1). Patients with wide resection margins had smaller tumors (median size, 2.73 cm vs 3.27 cm, respectively; *p* = 0.001) which were more frequently located in the left lateral section (27.7% vs 9.2%, respectively, *p* < 0.001) compared with those with narrow resection margins. No difference in pathological tumor differentiation, MVI, satellite nodules, or liver capsular involvement was observed between the two groups (*p* values, 0.11–0.58).

**Table 1:** Characteristics of the included patients

Characteristic	Narrow margin group				Wide margin group	*p* value^b^
	All, (*n* = 282)	Training dataset, (*n* = 198)	Testing dataset, (*n* = 84)	*p* value^a^	(*n* = 137)	
Patient demographics						
Age, years	55 (46–63)	54 (45.75–63)	55 (49–62.75)	0.51	54 (47–62)	0.68
Sex, male	244 (86.5)	174 (87.9)	70 (83.3)	0.31	117 (85.4)	0.76
Chronic hepatitis B, positive	225 (79.8)	165 (83.3)	60 (71.4)	**0.02**	108 (78.8)	0.82
Radiological cirrhosis, Positive	201 (71.3)	141 (71.2)	60 (71.4)	0.97	100 (73.0)	0.71
Pathological cirrhosis, Positive^c^	154 (58.1)	112 (58.6)	42 (56.8)	0.78	78 (59.5)	0.79
Laboratory parameters						
ALT, IU/L	33 (22.75–47.25)	33 (23–48)	31.5 (21.25–46.25)	0.84	32 (23–45)	0.64
AST, IU/L	31 (24–42)	30 (24.75–42.00)	32 (24.00–43.75)	0.85	28 (22.5–35.5)	**0.01**
Albumin, g/dL	43.95 (40.78–46.73)	44.05 (40.78–47.00)	42.85 (40.53–46.48)	0.33	44.3 (42.05–46.75)	0.17
Total bilirubin, μmol/L	14.3 (10.60–19.28)	14.4 (10.75–18.15)	14.3 (10.50–20.65)	0.81	13.1 (9.6–17.4)	0.10
ALBI score	−2.97 (−3.23 to −2.70)	−2.99 (−3.24 to −2.71)	−2.95 (−3.17 to −2.68)	0.24	−3.02 (−3.25 to −2.84)	0.08
Prothrombin time, s	11.6 (11.15–12.50)	11.6 (11.2–12.6)	11.6 (11.1–12.5)	0.61	11.4 (10.9–12.2)	**0.01**
Tumor characteristics						
Tumor size, cm	3.27 (2.17–5.57)	3.23 (2.13–5.48)	3.35 (2.34–5.59)	0.52	2.73 (1.80–3.90)	**0.001**
≤ 2 cm	61 (21.6)	47 (23.7)	14 (16.7)	0.19	43 (31.4)	**0.03**
2–5 cm	134 (47.5)	90 (45.5)	44 (52.4)	0.29	78 (56.9)	0.07
> 5 cm	87 (30.9)	61 (30.8)	26 (31.0)	0.98	16 (11.7)	**<** **0.001**
Tumor location^d^						**<** **0.001**
Caudate lobe	2 (0.7)	1 (0.5)	1 (1.2)	0.51	1 (0.7)	
Left lateral section	26 (9.2)	21 (10.6)	5 (6.0)	0.21	38 (27.7)	
Medial section	126 (44.7)	86 (43.4)	40 (47.6)	0.52	50 (36.5)	
Right posterior section	97 (34.4)	67 (33.8)	30 (35.7)	0.76	37 (27.0)	
Cross-regional involvement	31 (11.0)	23 (11.6)	8 (9.5)	0.61	11 (8.0)	
Tumor differentiation, grade 3–4^c^	87 (31.2)	65 (33.0)	22 (26.8)	0.31	37 (27.2)	0.41
MVI, present^c^	88 (38.9)	63 (39.6)	25 (37.3)	0.75	42 (35.9)	0.58
Satellite nodules, present^c^	9 (3.5)	6 (3.4)	3 (3.9)	0.73	6 (4.8)	0.58
Hepatic capsular involvement, positive^c^	96 (35.8)	65 (34.0)	31 (40.3)	0.34	59 (44.0)	0.11
Preoperative data						
Resection extent, major resection^c^	44 (16.1)	33 (17.10)	11 (13.8)	0.49	21 (15.7)	0.91
Type of operation, minimally invasive	112 (39.7)	75 (37.9)	37 (44.0)	0.33	71 (51.8)	**0.02**
Operative blood loss, mL^c^	200 (100–300)	200 (100–300)	200 (100–400)	0.42	100 (50–200)	**0.01**
Preoperative blood transfusion, yes^c^	16 (5.8)	11 (5.6)	5 (6.3)	0.85	2 (1.5)	0.05
Operative time, > 3 h	113 (41.4)	80 (41.0)	33 (41.3)	0.97	47 (35.3)	0.27
hospitalization time, days	8 (7–11)	8 (7–11)	8.5 (7–10)	0.41	7 (7–9)	**0.002**
Postoperative adjutant therapy, yes	55 (19.5)	39 (19.7)	16 (19.0)	0.90	29 (21.2)	0.69
Findings at recurrence						
Follow-up time, months	33.87 (19.85–47.95)	33.98 (19.68–48.21)	32.20 (20.15–47.18)	0.53	29.5 (17.58–44.50)	0.15
Early recurrence location				0.99		**0.01**
Without early recurrence	191 (67.7)	134 (67.7)	57 (67.9)		112 (81.8)	
Intrahepatic recurrence	59 (20.9)	42 (21.2)	17 (20.2)		18 (13.1)	
Macrovascular invasion	4 (1.4)	3 (1.5)	1 (1.2)		0 (0.0)	
Extrahepatic metastases	10 (3.5)	7 (3.5)	3 (3.6)		5 (3.6)	
Multiple metastases	18 (6.4)	12 (6.1)	6 (7.1)		2 (1.5)	

Continuous variables are presented as median, with interquartile range in parentheses, and categorical variables are the number of patients, with percentages in parentheses. Bold values denote statistical significance at the *p* < 0.05 level

^a^ Differences were computed by comparing dates between the training and testing dataset

^b^ Differences were computed by comparing data between patients with narrow-margin or wide-margin

^c^ Date were presented for patients who had complete documentation

^d^ Caudate lobe refers to segments 1; the left lateral section refers to segments 2, 3; the medial section refers to segments 4, 5, and 8; and the right posterior section refers to segments 6 and 7. Cross-regional involvement implies the involvement of more than one of the aforementioned locations

During a median follow-up time of 32.1 months, overall recurrence and early recurrence occurred in 35.3% (148/419) and 27.7% (116/419) of patients, respectively. Among the latter, 66.4% (77/116) exclusively developed intrahepatic recurrence, 3.4% (4/116) exclusively developed macrovascular invasion, 12.9% (15/116) exclusively developed extrahepatic metastases, and 17.2% (20/116) had recurrent tumors involving multiple sites. Patients with wide resection margins had higher rates of early RFS (78.3% vs 64.4%, respectively; *p* = 0.003) and overall RFS (60.6% vs 34.4%, respectively; *p* = 0.002) than their counterparts (Supplementary Fig. 1).

### Development of the MARGIN score

Based on patients with narrow resection margins, 13 preoperative variables (four clinical and nine MRI variables) were associated with early RFS rate at univariable Cox regression analyses (*p* values, 0.001–0.041, Table 2 and Supplemental Table 3). After accounting for collinearity, seven independent factors were further analyzed (Table 2).

**Table 2:** Uni- and multivariable Cox regression analyses for early RFS based on the training dataset

Variable	Univariate analyses			Multivariate analyses		
	β	Hazard ratio	*p* value	β	Hazard ratio	*p* value
Preoperative clinical and laboratory variables						
Age	− 0.03	0.97 (0.95–0.99)	0.007	− 0.03	0.97 (0.95–0.99)	0.005
AFP > 400 ng/mL	0.82	2.28 (1.36–3.79)	0.002	0.57	1.77 (1.03–3.03)	0.04
Vitamin K absence-II > 200 mAU/mL	1.01	2.76 (1.67–4.57)	< 0.001	0.87	2.38 (1.39–4.05)	0.002
The BCLC stage (A vs 0)	0.74	2.10 (1.07–4.12)	0.03	–	–	–
Preoperative imaging variables						
Tumor size, cm	0.13	1.14 (1.08–1.20)	< 0.001	–	–	–
Radiological involvement of liver capsule (present vs absent)	0.85	2.35 (1.33–4.13)	0.003	0.86	2.36 (1.32–4.24)	0.004
Intratumoral artery (present vs absent)	1.02	2.76 (1.69–4.52)	< 0.001	–	–	–
Nonperipheral washout (present vs absent)	0.77	2.17 (1.03–4.55)	0.04	–	–	–
Portal venous phase peritumoral hypo-enhancement (present vs absent)	0.98	2.66 (1.58–4.47)	< 0.001	–	–	–
Mosaic architecture (present vs absent)	0.83	2.30 (1.39–3.83)	0.001	–	–	–
Infiltrative appearance (present vs absent)	1.55	4.70 (2.01–11.03)	< 0.001	1.29	3.64 (1.43–9.29)	0.007
Blood products in mass (present vs absent)	0.63	1.88 (1.15–3.10)	0.01	–	–	–
Necrosis or severe ischemia (present vs absent)	0.64	1.89 (1.16–3.10)	0.01	–	–	–

Unless stated otherwise, data in parentheses are 95% confidence intervals

After accounting for multicollinearity, seven preoperative variables, including (1) age, (2) AFP > 400 ng/mL, (3) vitamin K absence-II > 200 mAU/mL, (4) tumor size, (5) radiological involvement of liver capsule, (6) nonperipheral washout, and (7) infiltrative appearance were analyzed at the multivariable analysis

*HR* hazard ratio, *Ref* reference

**Fig. 2:**
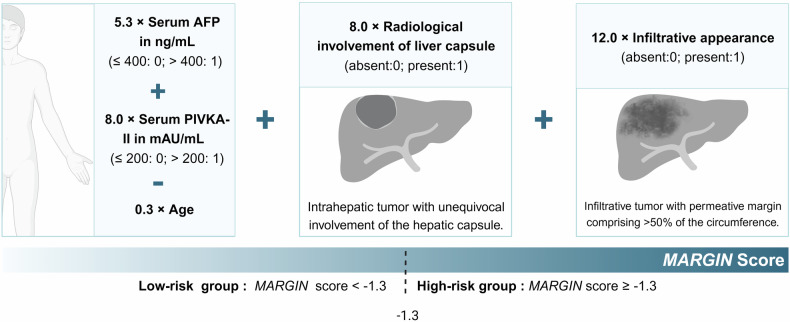
Graphical illustration of the *MARGIN* score

In the multivariable analysis, five variables were associated with early RFS, including age (HR = 0.97; 95% confidence interval [CI]: 0.95–0.99; *p* = 0.005), preoperative AFP > 400 ng/mL (HR = 1.77; 95% CI: 1.03–3.03; *p* = 0.04), protein induced by vitamin K absence or antagonist-II (PIVKA-II) > 200 mAU/mL (HR = 2.38; 95% CI: 1.39–4.05; *p* = 0.002), radiological involvement of liver capsule (HR = 2.36; 95% CI: 1.32–4.24; *p* = 0.004), and presence of an infiltrative appearance (HR = 3.64; 95% CI: 1.43–9.29; *p* = 0.01). These predictors were incorporated into the *MARGIN* score as follows (Fig. 2).

The *MARGIN* score = 5.3 × serum AFP in ng/mL (> 400, 1; ≤ 400, 0) + 8.0 × serum PIVKA-II in mAU/mL (> 200, 1; ≤ 200, 0) – 0.3 × age + 8.0 × radiological involvement of liver capsule (absent, 0; present, 1) + 12.0 × infiltrative appearance (absent, 0; present, 1).

The inter-rater agreement was excellent for the MARGIN score (intraclass correlation coefficient, 0.882; 95% CI: 0.863–0.899) and for the MARGIN risk strata (Fleiss κ value, 0.827; 95% CI: 0.748–0.906). Supplemental Table 4 summarizes the frequencies and inter-rater agreement of evaluated MR features.

### Validation of the MARGIN score

The *MARGIN* score demonstrated a *C*-index of 0.74 (95% CI: 0.68–0.80) in the training dataset and 0.75 (95% CI: 0.65–0.84) in the testing dataset. The td-AUCs of the *MARGIN* score in predicting 1- and 2-year RFS were 0.81 (95% CI: 0.73–0.88) and 0.72 (95% CI: 0.63–0.80) for the training dataset, and 0.78 (95% CI: 0.63–0.93) and 0.71 (95% CI: 0.57–0.85) for the testing dataset, respectively. Supplementary Fig. 2 illustrates the 2-year calibration and decision curves.

### Recurrence risk stratification

**Fig. 3:**
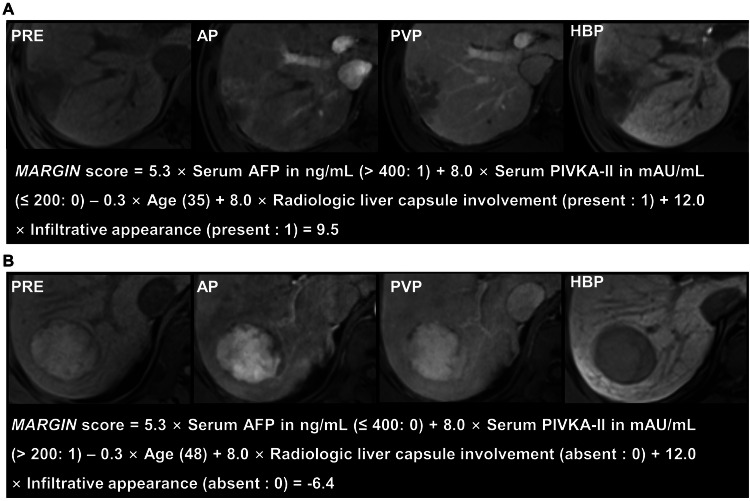
Contrast-enhanced MRIs of high-risk (**A**) and low-risk (**B**) patients with narrow resection margins. **A** A 35-year-old man with a 4.3 cm single HCC in segments VI and VII. Serum AFP was over 1210 ng/mL, and PIVKA-II was 141 mAU/mL. The tumor shows an infiltrative appearance and involvement of the liver capsule on MRI. This patient was classified as high-risk based on a *MARGIN* score of 9.5. The patient underwent a narrow resection margin and experienced recurrence at 2.1 months. **B** A 48-year-old man with a 5.2 cm HCC located in the segment VI and VII. The patient had a serum AFP level of 192 ng/mL and a PIVKA-II level of 1217 mAU/mL. The tumor does not show radiologic involvement of the liver capsule or infiltrative appearance. Based on a *MARGIN* score of − 6.4, the patient was categorized as low-risk. The patient received a narrow resection margin and remained recurrence-free during a 40.2-month follow-up. HCC, hepatocellular carcinoma; AFP, alpha-fetoprotein; PIVKA-II, protein induced by vitamin K absence or antagonist-II; PRE, pre-contrast phase; AP, arterial phase; PVP, peritumoral portal venous phase; HBP, hepatobiliary phase

In the training dataset, the *MARGIN* score ranged from − 22.2 to 24.3. Patients were classified as low- (*MARGIN* score < − 1.3) or high-risk (*MARGIN* score ≥ − 1.3). Figure 3 illustrates preoperative MRI images of representative patients from various risk profiles.

**Fig. 4:**
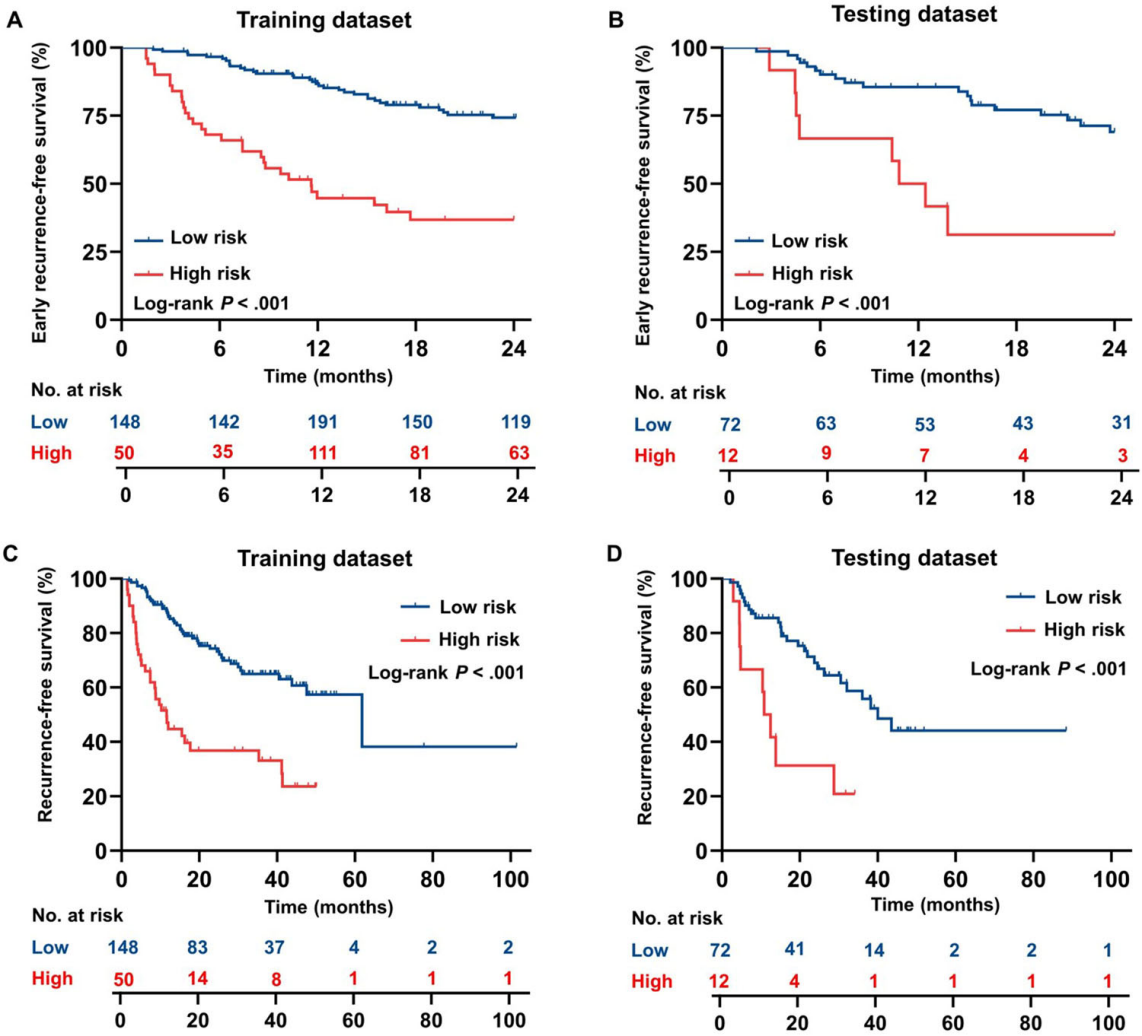
Results for early (≤ 2 years) RFS and overall RFS for high-risk (*MARGIN* score ≥ − 1.3 points) and low-risk (*MARGIN* score < − 1.3 points) individuals. For patients with narrow resection margins, Kaplan–Meier curves for early RFS (**A**, **B**) and overall RFS (**C**, **D**) are plotted

For patients with narrow resection margins, the high-risk group stratified by *MARGIN* (≥ − 1.3 points) had a lower cumulative rate of early RFS (both datasets *p* < 0.001) and overall RFS (both datasets *p* < 0.001, Fig. 4) at different time points than the low-risk group. To shed light on the biological underpinnings of MARGIN, known prognostic histopathologic features were compared between risk groups and presented in Supplemental Material 4 and Supplement Table 5.

### Application of MARGIN in informing the benefit of wide resection margins

After propensity score matching, 137 pairs of patients were matched for the narrow and wide resection margin groups, with standardized mean difference values ranging from 0.015 to 0.099. The characteristics of the propensity-score-matched cohorts are summarized in Supplement Table 6.

**Fig. 5:**
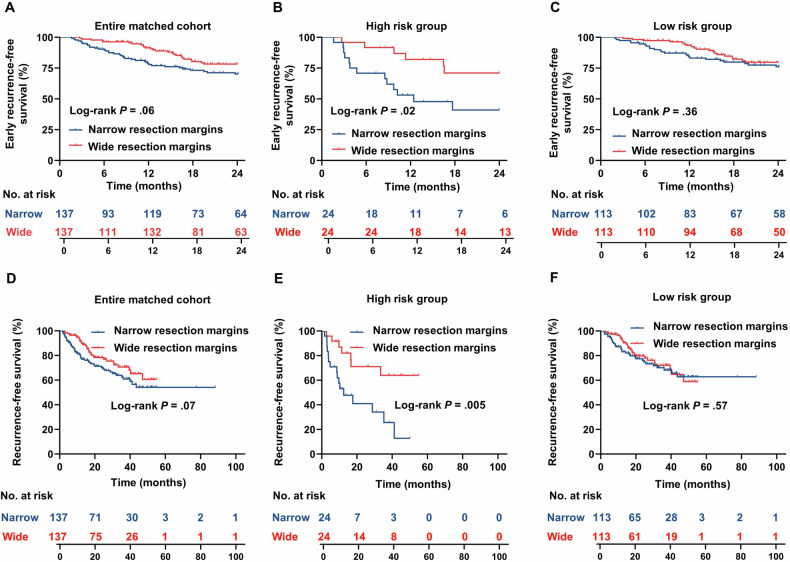
Survival outcomes of the entire propensity score-matched cohort, the high-risk group (MARGIN score ≥ − 1.3 points), and the low-risk group (MARGIN score ≤ − 1.3 points). Survival outcomes are plotted as Kaplan–Meier curves for early (≤ 2 years) recurrence-free survival (**A**–**C**) and overall recurrence-free survival (**D**–**F**)

For the entire matched cohort, no difference in early RFS rate was observed between patients with wide or narrow resection margins (78.3% vs 70.1%; HR = 0.62; 95% CI: 0.37–1.01; *p* = 0.06, Fig. 5A). However, after risk stratification based on *MARGIN* score, wide resection margins were associated with higher early RFS rate in the high-risk group (71.1% vs 41.0%; HR = 0.35; 95% CI: 0.14–0.87; *p* = 0.02, Fig. 5B), but not in the low-risk group (79.7% vs 76.1%; HR = 0.75; 95% CI: 0.41–1.37; *p* = 0.36, Fig. 5C). Similar results were observed in the subgroup analysis of patients with tumors of 2–5 cm. Specifically, a wide resection margin was associated with an improved early RFS rate in the high-risk group (80.5% vs 25.0%; HR = 0.16; 95% CI: 0.03–0.78; *p* = 0.02), but not in the low-risk group (78.4% vs 76.0%; HR = 0.76; 95% CI: 0.34–1.69; *p* = 0.50). For high-risk patients, margin width was the only prognostic factor for early RFS rate in the Cox regression analysis (HR = 0.34; 95% CI: 0.13–0.91; *p* = 0.03; Table 3).

**Table 3:** Univariable Cox regression analyses for early RFS in *MARGIN*-predicted high-risk (MARGIN score ≥ − 1.3 points) patients

Variable	Univariate analyses	
	Hazard ratio	*p* value
Preoperative clinical and laboratory variables		
Sex, male	0.35 (0.12–1.06)	0.06
Platelet count (^9/L)	1.00 (1.00–1.01)	0.32
Prothrombin time, s	1.17 (0.81–1.69)	0.40
AST, IU/L	1.00 (0.98–1.02)	0.21
ALT, IU/L	0.99 (0.97–1.01)	0.46
AST/ALT	3.82 (0.70–20.90)	0.12
Albumin, g/dL	1.03 (0.97–1.11)	0.32
Total bilirubin, umol/L	0.96 (0.90–1.04)	0.32
Child-Pugh class (B vs A)	3.69 (0.82–16.57)	0.09
ALBI score	0.59 (0.26–1.33)	0.21
ERASL-pre score	1.14 (0.47–2.73)	0.77
Tumor size, cm	1.02 (0.92–1.19)	0.34
Tumor size ≤ 2 cm	Ref	Ref
Tumor size 2–5 cm	0.55 (0.07–4.56)	0.58
Tumor size > 5 cm	1.22 (0.15–9.76)	0.85
The BCLC stage (A vs 0)	1.24 (0.16–9.50)	0.84
Pathological variables		
Pathological liver cirrhosis, positive^a^	1.08 (0.44–2.66)	0.87
Hepatic capsular involvement, positive^a^	1.04 (0.39–2.74)	0.94
Macrovascular invasion, present^a^	1.70 (0.67–4.29)	0.26
Tumor differentiation, grade 3–4^a^	1.60 (0.65–3.93)	0.31
Satellite nodules, present^a^	1.46 (0.19–10.97)	0.71
Treatment options		
Margin width, wide-margin	0.34 (0.13–0.91)	**0.03**
Resection extent, major resection^a^	0.96 (0.34–2.74)	0.94
Type of operation, minimally invasive	0.73 (0.30–1.80)	0.50
Postoperative adjuvant therapy, yes^a^	1.23 (0.34–4.44)	0.75

Unless stated otherwise, data in parentheses are 95% CIs. Bold values denote statistical significance at the *p* < 0.05 level

*HR* hazard ratio, *Ref* reference

^a^ Date was presented for patients who had complete documentation

Likewise, based on the entire matched cohort, no difference in overall RFS rate was observed between patients with wide or narrow resection margins (60.6% vs 54.1%, respectively; HR = 0.67; 95% CI: 0.43–1.03; *p* = 0.07, Fig. 5D). After risk stratification based on *MARGIN* score, wide resection margins were associated with improved overall RFS rate in the high-risk group (64.0% vs 12.8%; HR = 0.30; 95% CI: 0.13–0.69; *p* = 0.01, Fig. 5E), but not in the low-risk group (58.8% vs 62.8%; HR = 0.86; 95% CI: 0.51–1.45; *p* = 0.57, Fig. 5F). For high-risk patients, margin width was also the only prognostic factor for overall RFS rate at the Cox regression analysis (HR = 0.29; 95% CI: 0.12–0.72; *p* = 0.007; Supplement Table 7). The incremental value of the MARGIN score to ERASL-pre is shown in Supplemental Fig. 3 and Supplemental Material 5.

## Discussion

Based on 282 patients with curative-intent narrow resection margins, we developed a prognostic score named “*MARGIN*” to predict the early RFS rate. Utilizing five preoperative features, the *MARGIN* score achieved a *C*-index of 0.75 in the testing dataset, effectively stratifying patients into prognostically distinct high- (*MARGIN* score ≥ − 1.3) and low-risk (*MARGIN* score < − 1.3) groups. When applied to 137 pairs of propensity-score-matched patients, wide resection margins were associated with an improved early RFS rate for the *MARGIN*-predicted high-risk patients (*p* = 0.02), but not for the low-risk ones (*p* = 0.36).

The radicality of HCC resection is influenced by surgical choice and directly affects survival outcomes [1]. Wide resection margins have been widely correlated with a lower risk of early recurrence compared with narrow resection margins [4, 5, 10], which aligns with our findings in all enrolled patients (early RFS rate, 78.3% vs 64.4%; *p* = 0.003). However, several factors (i.e., tumor size, tumor location and liver function [9, 28, 29]) may influence margin width selection, as well as survival outcomes, leaving it unclear whether a wide resection margin is a surrogate of underlying tumor biology or truly a technical issue that independently affects survival. Notably, after propensity-score matching, no difference in early RFS rate was detected between the wide and narrow resection margin groups (78.3% vs 70.1% *p* = 0.06), indicating that the prognostic significance of margin width varied across patient subpopulations. Given the uncertainties and potential impairments caused by improper resections, adopting a benefit-based tailored treatment strategy will prove to be more effective than a blanket “treat all” approach.

The *MARGIN* score allows for better estimates of an individual patient’s likelihood of benefiting from wide resection margins. Among propensity-score matched patients, an improved early RFS rate was observed for wide resection margins in the *MARGIN* high-risk patients (71.1% vs 41.0%; *p* = 0.02), but not for the low-risk ones (79.7% vs 76.1%; *p* = 0.36). Additionally, for patients with *MARGIN*-predicted high-risk status, margin width was a significant predictor of early recurrence, even after adjusting for established prognostic factors. *MARGIN* also enabled the identification of patients who were more likely to benefit from wide resection margins within the ERASL-predicted low-risk category, highlighting the incremental prognostic value of the *MARGIN* to the ERASL which is a widely validated post-resection predicting score.

Furthermore, our multivariable risk prediction is more effective than conventional “one-variable” subgroup analyses, as it accounts for potential interactions and confounding effects of other variables. The *MARGIN* score was constructed based on comprehensive clinic-radiological features (including 11 clinical and 51 radiologic features for each patient) with rigorous modeling methodologies, making it more relevant to individual patients than recommendations based on coarse groupings of large numbers of heterogeneous patients [30]. Notably, the *MARGIN* score was exclusively developed for patients with BCLC 0 or A HCC and well-preserved liver function, whose prognosis is well-known in relation to surgical choice [1, 22]. Despite being the optimal surgical candidate as recommended by major guidelines, whether *MARGIN* could be extrapolated to inform the selection of wide or narrow resection margin in patients with tumors beyond a single BCLC 0/A stage (e.g., multinodular BCLC A tumor) remains to be elucidated.

The *MARGIN* score emphasized the potential of imaging features (i.e., infiltrative appearance and radiologic involvement of the liver capsule) in assisting individualized treatment. Specifically, infiltrative appearance is a well-established adverse prognostic feature of HCC and has been incorporated into the most recent BCLC staging system [31]. The infiltrative appearance has been associated with poorer differentiation, more frequent MVI, reduced capsule formation, and higher expression of stemness-related markers on pathology [32]. For infiltrative HCC, wide resection margins have been related to a survival benefit compared to narrow resection margins [17]. Radiologic involvement of the liver capsule was also predictive of early recurrence, along with narrow resection margins, potentially due to larger tumor size and increased capsular spread [33]. Overall, the *MARGIN* score can effectively profile tumor biologic aggressiveness. In particular, *MARGIN*-predicted high-risk status was associated with more frequent MVI, poorer differentiation, and liver capsule involvement in pathology.

The *MARGIN* score was derived from semantic imaging features that are user-friendly. Beyond semantics, several studies have used quantitative radiomics to predict post-resection outcomes in HCC [34, 35]. However, the complexity of radiomic analysis requires specialized software and expertise, making it less accessible for clinical adoption. Notably, despite an excellent inter-rater agreement for *MARGIN* and *MARGIN*-defined risk groups, a suboptimal agreement was found for *MARGIN*-included imaging features. The inadequate agreement for imaging features may have been inherent to subjective manual assessment, which was consistent with previous studies [36]. These results highlight the need to enhance the reproducibility of imaging assessment, possibly through standardized lexicons and objective criteria.

This study had several limitations. First, it was a single-center study with a relatively small sample size. Therefore, no external validation was available to test and refine the *MARGIN* score. Second, up to 79.4% of the included patients had chronic hepatitis B. Therefore, our model may not be applicable to patients with non-hepatitis B etiology. Third, our database did not include information on whether liver resections were anatomical. Although Liu et al [37] demonstrated wide resection margins are more important than anatomical resection for prognosis in patients with MVI-positive HCC, we were unable to determine which factor, anatomical resection or wide resection margins, had a greater influence on survival results. Therefore, further prospective multi-center studies are warranted to validate our findings, ideally in the setting of clinical trials.

In conclusion, in patients who underwent curative-intent resection for single BCLC 0 or A HCC, we developed and validated a prognostic score called the *MARGIN* for early recurrence based on five preoperative clinical and MRI features. Wide resection margins were associated with improved early RFS rate for the *MARGIN*-predicted high-risk patients, but not for the low-risk ones.

## Supplementary information

ELECTRONIC SUPPLEMENTARY MATERIAL

## Abbreviations

AFP: Alpha-fetoprotein
BCLC: Barcelona clinic liver cancer
HCC: Hepatocellular carcinoma
MRI: Magnetic resonance imaging
MVI: Microvascular invasion
PIVKA-II: Protein induced by vitamin K absence or antagonist-II
RFS: Recurrence-free survival
