# Supplementary material for: MRI-based prediction of the need for wide resection margins in patients with single hepatocellular carcinoma
Source: Eur Radiol. 2024 Sep 5;35(4):1772–84. doi: 10.1007/s00330-024-11043-5 (PMC11913993; doi:10.1007/s00330-024-11043-5)

MRI-based Prediction of the Need for Wide Resection Margins in Patients with Single Hepatocellular Carcinoma

ELECTRONIC SUPPLEMENTARY MATERIAL

Supplemental Material 1 Demographic, Laboratory, Surgical, and Histologic data collection

Demographic information and laboratory data within two weeks before liver resection were collected from electronic patient records. The type of operation (minimally invasive or open), resection extent (major or minor), and resection margin width were decided at the surgeons' discretion after considering patient performance status, comorbidities, liver function, estimated future liver remnant volume, tumor location, and tumor proximity to major vasculatures. Information regarding surgical procedures was collected, including the duration of the procedure, intraoperative blood loss, and transfusion. Major resection was defined as the removal of ≥3 Couinaud segments, while minor resection involved<3 segments [1].

Histopathological information on resection margin width, tumor differentiation, satellite nodules, MVI, and liver capsular involvement were retrieved from the routine pathology report. The resection margin width was defined as the minimal macroscopic distance between the tumor edge and the surgical transection plane. In cases where satellite nodules were present, the tumor nodule closest to the surgical transection plane was analyzed [2]. Based on resection margin width, the study population was then categorized into either the wide- (≥1 cm) or narrow-resection margin (<1 cm) group, following guidelines [3, 4] and results of a prior randomized prospective experiment [1].

References

1. Shi M, Guo RP, Lin XJ et al. Partial hepatectomy with wide versus narrow resection margin for solitary hepatocellular carcinoma: a prospective randomized trial. Ann Surg 2007; 245 (1): 36-43.

2. Zhou Z, Qi L, Mo Q et al. Effect of surgical margin on postoperative prognosis in patients with solitary hepatocellular carcinoma: A propensity score matching analysis. J Cancer 2021; 12 (15): 4455-4462.
3. Abdo AA, Hassanainh M, AlJumah A et al. Saudi Guidelines for the Diagnosis and Management of Hepatocellular Carcinoma: Technical Review and Practice Guidelines. Ann Saudi Med 2012; 32 (2): 174-199.
4. Wang CK, Grp S, Grp D et al. Management consensus guideline for hepatocellular carcinoma: 2016 updated by the Taiwan Liver Cancer Association and the Gastroenterological Society of Taiwan. J Formos Med Assoc 2018; 117 (5): 381-403.

Supplemental Material 2. MR examinations and Analysis.

Contrast-enhanced MR images of each patient were acquired using either extracellular contrast-enhanced MRI (ECA-MRI) or gadoxetate disodium-enhanced MRI (EOB-MRI) on one of the seven types of 3.0 T or 1.5 T MR systems (Siemens MAGNETOM Skyra 3.0 T; Siemens TrioTim 3.0 T; Siemens Avanto 1.5 T; GE SIGNA™ Architect 3.0 T; GE SIGNA™ Premier 3.0 T; GE Discovery MR 750 3.0 T; Philips Ingenia Elition X 3.0 T; uMR588 1.5 T). ECA-MRI was performed using 0.1 mmol/kg gadopentetate dimeglumine (Magnevist®; Bayer Schering Pharma AG, Berlin, Germany), gadoterate meglumine (Dotarem®; Guerbet, Paris, France), or gadobenate dimeglumine (MultiHance®; Bracco, Shanghai, China). 0.025 mmol/kg EOB (Primovist®; Bayer Schering Pharma AG, Berlin, Germany or XianAi®; Chia Tai TianQing Pharmaceutical Co., Ltd, Jiangsu, China) was injected at a rate of 1.0-2.0 ml/s for EOB-MRI acquisition.

Before MR exams, all patients were asked to fast for 6-8 hours. The MRI sequences included: (***a***) T2-weighted imaging; (***b***) diffusion-weighted imaging (b values: 0, 50, 500, 800, 1000, and 1200 s/mm^2 [Siemens MAGNETOM Skyra]; 0, 50, 800 s/mm^2 [Siemens TrioTim; Siemens Avanto]; 0, 50, 1000 s/mm^2 [Siemens Avanto; GE SIGNA™ Architect; GE SIGNA™ Premier; uMR588]; 0, 200, 800, and 1000

s/mm^2 [GE Discovery MR 750]; 0, 200, 1000 s/mm^2 [Philips Ingenia Elition X]) with apparent diffusion coefficient maps reconstructed using the monoexponential model; (***c***) in- and opposed-phase T1-weighted imaging; and (***d***) dynamic T1-weighted imaging in the pre-contrast phase, late arterial phase, portal venous phase (60 s after the start of contrast media injection), delayed phase (for ECA-MRI, 3min after start of contrast media injection) or transitional phase (for EOB-MRI, 5min after the start of contrast media injection), and hepatobiliary phase (for EOB-MRI, 20min after the start of contrast media injection). The arterial phase images were acquired with either a bolus-tracking method (acquisition triggered 7s after the arrival of the contrast bolus in the celiac trunk) or a multiple arterial phase imaging technique (acquired with an 18 s-breath-hold 20s after the start of contrast media injection and further reconstructed with a temporal resolution of 3 s).

All deidentified images were reviewed independently by three fellowship-trained abdominal radiologists (with 7, 10, and 3 years of experience in liver imaging), who were aware that the patients had HCC but blinded to the clinical and follow-up information. Inter-rater disagreements for binary features were then resolved with the majority interpretation, while disagreements for ordinal/categorical features were resolved either with the majority interpretation or by consulting a senior abdominal radiologist with over 20 years of experience in liver MRI.

Supplemental Material 3. Statistical Analysis.

Briefly, the missing data was computed via multiple imputations through chained equations, which comprised 0.7% to 5.2% of the laboratory variables. Survival outcomes were estimated with the Kaplan-Meier method and compared using the log-rank test. The interobserver agreement was assessed using Fleiss' κ value for binary variables, the intraclass correlation coefficient (ICC) for continuous variables, and the weighted κ value for ordinal/categorical variables. Agreement was considered poor (κ or ICC < 0.2), fair (κ or ICC: 0.2-0.4), moderate (κ or ICC: 0.4-0.6), substantial (κ or ICC: 0.6-0.8), or excellent (κ or ICC > 0.8).

Model Development and Validation

The assumption made was that achieving acceptable oncologic outcomes with narrow resection margins would reduce the necessity for wide resection margins. Based on this premise, a prognostic score called MARGIN was developed to estimate early RFS rate in patients with narrow resection margin, serving as the basis for predicting the need for wide resection margin.

To ensure effective multivariable regression analyses, the sample size was estimated for at least five outcome events per variable (1). Eligible patients with narrow resection margins were randomly divided into training and testing datasets at a ratio of 7:3. In the training dataset, predictive preoperative demographic, laboratory, and MRI variables were identified using univariable and multivariable Cox regression analyses with backward step-wise selection and five-fold cross-validation (detailed in Supplemental Table 3). Inter-variable collinearities were calculated by the Spearman's correlation analysis. In cases of collinearity (rho>0.6), the variable with the smallest *P* value was retained. A scoring system was formulated based on the statistically significant variables from the multivariable Cox analysis weighted by their respective β coefficients, with the largest β coefficient scaled as 12 points and the remaining proportionally rounded to the nearest integers to improve clinical utility.

Model discrimination was evaluated with the concordance index (C-index) and the time-dependent area under the curve (time-dependent AUC). Model calibration was assessed by calibration curve. Decision curves were plotted to estimate the score' clinical usefulness by quantifying the net benefits at different threshold probabilities. To divide patients into high- and low-risk groups for early recurrence, the optimal threshold value of the score was determined at its 75th percentile based on the training dataset.

Model utility in informing the need for wide resection margins

The models' effectiveness in determining the need for wide resection margin was evaluated in a propensity score-matched cohort. Specifically, a propensity score utilizing logistic regression was created based on four parameters (i.e., cirrhosis, the ALBI score, tumor size, and tumor location), which have been shown to significantly affect resection margin selection and recurrence risk (2,3,4). Patients who received

wide resection margins were matched with those who underwent narrow resection margins using a one-to-one optimal pair matching method based on the propensity score. The matched data were compared using paired *t* tests or Wilcoxon signed-rank tests for continuous variables and McNemar tests or stratified logistic regression for categorical variables. Subgroup analyses were performed for *MARGIN*-predicted high-risk patients with tumor sizes ranging from 2-5 cm.

Uni- and multivariable Cox regression analyses were conducted to adjust for other previously-reported prognostic factors. Furthermore, the matched patients were categorized relative to the preoperative Early Recurrence After Surgery for Liver Tumor (ERASL-pre) score, which was derived from similar patient populations, to evaluate whether the *MARGIN* score had incremental prognostic value to ERASL-pre.

R (version 4.2.2; R Foundation for Statistical Computing, Vienna, Austria) was used for the statistical analysis. All statistical tests were two-sided, and $P<0.05$ was considered statistically significant.

References:

1. Moons KG, Altman DG, Reitsma JB et al. Transparent Reporting of a multivariable prediction model for Individual Prognosis or Diagnosis (TRIPOD): explanation and elaboration. Ann Intern Med 2015; 162 (1): W1-73.
2. Chan AWH, Zhong J, Berhane S et al. Development of pre and post-operative models to predict early recurrence of hepatocellular carcinoma after surgical resection. J Hepatol 2018; 69 (6): 1284-1293.
3. Ban D, Ogura T, Akahoshi K et al. Current topics in the surgical treatments for hepatocellular carcinoma. Ann Gastroent Surg 2018; 2 (2): 137-146.
4. de Faria LL, Darce GF, Bordini AL et al. Liver Surgery: Important Considerations for Pre- and Postoperative Imaging. Radiographics 2022; 42 (3): 722-740.

Supplemental Material 4. Pathological Characteristics between Risk Groups for Patients with Narrow Resection Margins.

The pathological characteristics of different risk groups for patients with narrow

resection margins are summarized in **Supplement Table 5**. Briefly, the high-risk group had more frequent MVI (61.3% vs. 24.8%; *P* <.001), poorly differentiated tumors (45.2% vs. 27.3%; *P* =.01), and liver capsular involvement (59.7% vs 31.1%; *P* <.001) than the low-risk group. No difference in satellite nodules was observed between the high- and low-risk groups (8.6% vs 2.8%; *P* =.06).

Supplemental Material 5. Incremental Prognostic Values of the MARGIN score to ERASL-pre

According to the ERASL-pre score for the matched cohort, 93.0% (255/274) of the patients were at low risk of early recurrence, 6.9% (19/274) at medium risk, and none at high risk. The ERASL-predicted low-risk patients could be further stratified by the *MARGIN* score, with 86.2% (220/255) classified as *MARGIN* low-risk and 13.7% (35/255) as *MARGIN high*-risk. Wide resection margins were associated with an improved early RFS rate for *MARGIN*-predicted high-risk group within the ERASL-predicted low-risk group (71.8% vs. 19.0%; HR=0.20, 95% CI, 0.06-0.63; *P*=.01, **Supplementary Figure 3A**), but not for the *MARGIN*-predicted low-risk within ERASL-predicted low-risk group (80.5% vs. 77.5%; HR=0.79, 95% CI, 0.43-1.48; *P*=0.47, **Supplementary Figure 3B**).

Supplemental Table 1. MR sequences and parameters.

Sequence	Fat suppression	TR (ms)	TE (ms)	Flip angle (°)	ST (mm)	Spacing (mm)	Matrix size	FOV (mm^2)	Acquisition Time (s)
Siemens MAGNETOM Skyra 3.0 T (18-channel body array coil)									
T2-weighted 2D FSE	Yes	2160	100	160	6	1.8	320×288	433×433	36
Diffusion-weighted imaging*	Yes	5600	68	90	6	1.8	100×76	380×289	233
In- and opposed-phase T1-weighted imaging	No	81	2.72/1.4	70	6	1.8	352×286	400×325	24
Dynamic T1-weighted 3D GRE	Yes	3.95	1.92	9	2.5	-	352×256	400×296	14
***Siemens TrioTim* 3.0 T (*8-channel body anterior coil*)**									
T2-weighted 2D FSE	Yes	2700	95	140	6	7.8	320×147	442×254	RS
Diffusion-weighted imaging*	Yes	5900	76	90	6	7.8	192×154	393×393	245
In- and opposed-phase T1-weighted imaging	No	181	2.2/3.67	65	6	7.8	256×131	410×269	18
Dynamic T1-weighted 3D GRE	Yes	3.47	1.25	9	2.4	-	320×133	434×257	17
Siemens Avanto 1.5 T (30-channel body anterior coil)									
T2-weighted 2D FSE	Yes	2530	84	150	6	7.8	256×187	293×251	47
Diffusion-weighted imaging*	Yes	3600	88	90	6	7.8	192×115	310×232	92
In- and opposed-phase T1-weighted imaging	No	72	4.92/2.22	70	6	7.8	256×158	328×225	16
Dynamic T1-weighted 3D GRE	Yes	5.41	2.39	10	2.5	-	320×138	382×238	15
Siemens Avanto 1.5 T (8-channel body anterior coil)									
T2-weighted 2D FSE	Yes	2710	84	150	7.5	9.75	256×177	308×380	27
Diffusion-weighted imaging*	Yes	2000	72	90	7.5	9.75	192×125	308×379	20
In- and opposed-phase T1-weighted imaging	No	87	4.92/2.22	70	7.5	9.75	256×187	308×380	33
Dynamic T1-weighted 3D GRE	Yes	5.4	2.38	10	2	-	320×131	241×407	15
***GE SIGNA™* Architect 3.0 T (*30-channel body anterior coil*)**									
T2-weighted 2D FSE	Yes	2400	85	111	7	2	320×192	380×304	34
Diffusion-weighted imaging*	Yes	5000	Minimum	90	7	2	160×128	380×342	RS
In- and opposed-phase T1-weighted imaging	No	233.8	2.3/1.1	55	7	2	160×288	380×323	18
Dynamic T1-weighted 3D GRE	Yes	3.9	1.7	15	3	-	320×240	380×380	15
GE SIGNA™ Premier 3.0 T (30-channel body anterior coil)									
T2-weighted 2D FSE	Yes	2200	85	111	7	2	320×224	304×380	47
Diffusion-weighted imaging*	Yes	5000	Minimum	90	7	2	120 × 240	380× 380	RS
In- and opposed-phase T1-weighted imaging	No	146.8	2.3/1.1	55	7	2	320×192	342×380	16
Dynamic T1-weighted 3D GRE	Yes	3.2	1.4	15	2.4	-	320×240	380× 380	15
GE Discovery MR 750 3.0 T (16-channel phased-array torsor coil)									
T2-weighted 2D FSE	Yes	6315	78	111	6	2	288×244	360×280	RS
Diffusion-weighted imaging*	Yes	9230	Minimum	90	6	2	128 × 128	360× 380	RS
In- and opposed-phase T1-weighted imaging	No	150	2.5/1.3	70	6	2	288×192	420×420	31
Dynamic T1-weighted 3D GRE	Yes	4.1	1.9	15	2	-	512×512	380× 300	15
Philips Ingenia Elition X 3.0 T (16-channel body anterior coil)									
T2-weighted 2D FSE	Yes	1883.51	90	90	6.8	8.5	272×78	239×69	46
Diffusion-weighted imaging*	Yes	1653.65	60.29	90	7	8.5	142×140	141×139	52
In- and opposed-phase T1-weighted imaging	No	164.53	2.30/1.15	50	6	7.5	256×201	206×162	11
Dynamic T1-weighted 3D GRE	Yes	4.2	0	10	3	1.5	344×252	303×222	13
uMR588 1.5 T (6-channel body anterior coil)									
T2-weighted 2D FSE	Yes	2600	99.2	90	6.5	1.5	256×168	427×320	39
Diffusion-weighted imaging*	Yes	3350	77	90	6.5	10	128×92	320×400	RS
In- and opposed-phase T1-weighted imaging	No	117.6	4.69/2.26	60	6.5	1.3	256×174	320×400	29
Dynamic T1-weighted 3D GRE	Yes	4.2	1.88	10	2.5	-	256×154	255×400	13

Note. —TR = repetition time; TE = echo time; ST = section thickness; FOV = field of view; 2D = two-dimensional; 3D = three-dimensional; FSE = fast spin-echo; GRE = gradient recall echo; RS = Respiratory gating. * Images were acquired under free breath.

Supplemental Table 2: Definitions of the imaging features.

Imaging features	Definition	MR Image sequence
Bio-oncological related features		
LI-RADS major features		
Nonrim arterial phase hyperenhancement (present vs. absent)	Nonrim-like enhancement of the tumor in the arterial phase unequivocally greater in whole or in part than the liver. (1,2)	Late arterial phase
Nonperipheral washout (present vs. absent)	Nonperipheral visually assessed temporal reduction in the enhancement of the tumor in whole or in part relative to composite liver tissue in the portal venous phase or delayed phase. (1,2)	Portal venous or delayed phase for extracellular contrast agents or gadobenate; portal venous phase for gadoxetate
Enhancing capsule (present vs. absent)	Smooth, uniform, sharp border around most or all of a tumor, unequivocally thicker or more conspicuous than fibrotic tissue around background nodules, and visible as enhancing rim in portal venous phase, delayed phase, or transitional phase. (1,2)	Portal venous, delayed, or transitional phase
Tumor size (cm)	Largest outer-edge-to-outer-edge dimension of a tumor. (1,2)	Pick the phase, sequence, plane in which the margins are clearest. Do not measure in arterial phase or DWI.
LI-RADS ancillary features		
Diffusion restriction (present vs. absent)	Signal intensity of the tumor higher than the liver on diffusion-weighted images not caused only by T2 shine-through. (1,2)	High b value (e.g., ≥800) DWI
Mild-moderate T2 hyperintensity (present vs. absent)	Signal intensity of the tumor on T2WI higher than the liver, similar to or lower than a non-iron-overloaded spleen, and lower than simple fluid. (1,2)	T2WI
Corona enhancement (present vs. absent)	Peritumoral enhancement in the late arterial phase or early portal venous phase. The enhancement is contiguous with and surrounds all or part of the tumor. (1,2)	Late arterial or early portal venous phase
Nonenhancing capsule (present vs. absent)	Subtype of capsule that does not show enhancement on any image. (1,2)	Multiple sequences
Nodule-in-nodule architecture (present vs. absent)	Presence of smaller inner nodule within and having different imaging features than larger outer nodule.	Multiple sequences
Mosaic architecture (present vs. absent)	Presence of any combination of internal nodules, compartments, or septations, within a solid or mostly solid mass. (1,2)	Multiple sequences
Blood products in mass (present vs. absent)	Intralesional or perilesional hemorrhage in the absence of biopsy, trauma, or intervention. (1,2)	Multiple sequences
Fat in mass, more than adjacent liver (present vs. absent)	Excess fat within a mass, in whole or in part, relative to the adjacent liver. (1,2)	In-/opposed phase; fat-suppressed images compared to non-fat-suppressed images with similar or identical weighting
Fat sparing in solid mass (present vs. absent)	Relative paucity of fat in solid mass relative to steatotic liver OR in inner nodule relative to steatotic outer nodule. (1,2)	In-/opposed phase; fat-suppressed images compared to non-fat-suppressed images with similar or identical weighting
Iron sparing in solid mass (present vs. absent)	Paucity of iron in solid mass relative to iron-overloaded liver OR in inner nodule relative to siderotic outer nodule. (1,2)	In-/opposed phase; T2WI
Transitional phase hypointensity (preset vs. absent)	Signal intensity of the tumor in the transitional phase unequivocally less, in whole or in part, than the liver. (1,2)	Transitional phase
Hepatobiliary phase hypointensity (preset vs. absent)	Signal intensity of the tumor in the hepatobiliary phase unequivocally less, in whole or in part, than the liver. (1,2)	Hepatobiliary phase
Marked T2 hyperintensity (present vs. absent)	Signal intensity of the tumor on T2WI higher than non-iron-overloaded spleen and as high as or almost as high as simple fluid. (1,2)	T2WI
Iron in mass, more than liver (present vs. absent)	Excess iron in a mass relative to the background liver. (1,2)	In-/opposed phase; T2WI
Parallels blood pool enhancement (present vs. absent)	Temporal pattern in which enhancement approximates blood pool in all phases. (1,2)	Post-contrast phases
Undistorted vessels (present vs. absent)	Vessels traversing a tumor without displacement, deformation, or other alteration. (1,2)	Post-contrast phases
LR-M features		
Rim arterial phase hyperenhancement		Late arterial phase

(present vs. absent)	Spatially defined subtype of arterial phase hyperenhancement in which arterial phase enhancement is most pronounced in tumor periphery. (1,2)	
Peripheral washout (present vs. absent)	Presence of apparent washout most pronounced in the tumor periphery. (1,2)	Portal venous or delayed phase for extracellular contrast agents or gadobenate; portal venous phase for gadoxetate
Delayed central enhancement (present vs. absent)	Central area of progressive postarterial phase enhancement. (1,2)	Postarterial phases
Targetoid restriction (present vs. absent)	Concentric pattern on diffusion-weighted imaging characterized by restricted diffusion in tumor periphery with less restricted diffusion in tumor center. (1,2)	DWI or ADC map
Targetoid transitional phase or hepatobiliary phase appearance (present vs. absent)	Concentric pattern in transitional or hepatobiliary phase characterized by moderate-to-marked hypointensity in the tumor periphery with milder hypointensity in the center. (1,2)	Transitional or hepatobiliary phase
Marked diffusion restriction (present vs. absent)	Signal intensity of the tumor similar to or higher than non-iron-overloaded spleen on diffusion-weighted images not caused only by T2 shine-through. (1,2)	High b value (e.g.,≥800) DWI
Infiltrative appearance (present vs. absent)	Infiltrative tumor with permeative margin comprising >50% of the circumference. (1,3)	Multiple sequences
Necrosis or severe ischemia (present vs. absent)	Presence of nonenhancing area in a solid mass, not attributable to a cystic component, prior treatment, or intralesional hemorrhage. (1,2)	Post-contrast phases and T2WI
Other tumor-related prognostic features		
Radiological involvement of liver capsule (present vs. absent)	Intrahepatic tumor with unequivocal involvement of the hepatic capsule.	Multiple sequences
T2-weighted peritumoral hyperintensity (present vs. absent)	Presence of irregular, wedge-shaped, or flame-like area adjacent to the tumor on T2WI of which the signal intensity is mildly or moderately higher than liver and similar to or less than a non-iron-overloaded spleen. (3,4)	T2WI
Portal venous phase peritumoral hypo-enhancement (present vs. absent)	Presence of irregular, wedge-shaped, or flame-like hypo-enhancing area adjacent to the tumor in the portal venous phase. (3,4,5)	Portal venous phase
Markedly low apparent diffusion coefficient value (present vs. absent)	Apparent diffusion coefficient value of the tumor similar or lower than that of a non-iron-overloaded spleen.	ADC map
Arterial phase hyperenhancement proportion (<50% vs. ≥50%)	The proportion of tumor volume demonstrating arterial phase hyperenhancement. (6)	Late arterial phase
Intratumoral artery (present vs. absent)	Presence of discrete arteries within the tumor on arterial phase images. (3,6,7)	Early or late arterial phase
Complete capsule (present vs. absent)	Presence of non-disrupted capsule in all imaging planes. (3,8)	Multiple sequences
Non-smooth tumor margin (present vs. absent)	The tumor margin is irregular and/or has areas of bulging, nodular projection, or infiltration into adjacent tissues at the tumor periphery in any imaging plane. (3,7,10)	Multiple sequences
Marked hepatobiliary phase hypointensity (present vs. absent)	Signal intensity of the tumor in the hepatobiliary phase lower than that of the liver and similar to or lower than that of intrahepatic vessels. (11)	Hepatobiliary phase
Hepatobiliary phase peritumoral hypointensity (present vs. absent)	Presence of irregular, wedge-shaped or flame-like hypointense area of liver parenchyma located outside of the tumor margin in the hepatobiliary phase. (3,4,11,12)	Hepatobiliary phase
Tumor growth subtype (13,14)		Multiple sequences
Single nodular type	A round expanding nodule with a distinct margin in all imaging planes.	
Single nodule type with extranodular growth	An expanding nodule with areas of bulging or nodular extranodular projection involving less than 50% of tumor circumference.	
Confluent multinodular type	A cluster of small and confluent nodules.	
Infiltrative type	Tumor with extranodular growth involving more than 50% of circumference.	
Pre-contrast T1-weighted hypointensity (present vs. absent)	Signal intensity of the tumor on pre-contrast T1-weighted imaging unequivocally lower than that of the liver.	Pre-contrast T1WI
Bilobar involvement (present vs. absent)	The tumor has invaded across both the right and left lobes of the liver.	Multiple sequences
Early peritumoral hyperenhancement (present vs. absent)	Nonmasslike area adjacent to a mass showing hyperenhancement in the early arterial phase.	Early arterial phase

Imaging features related to underlying liver condition		
The background liver-related features		
Radiologically-evident cirrhosis (present vs. absent)	Unequivocal morphological alterations of the liver, including surface nodularity, small liver volume, expansion of space between liver and anterior abdominal wall and perihilar, gallbladder fossa and ligamentum teres spaces, hypertrophy of caudate and/or left lateral section, atrophy of anterior right section and/or left medial section, anterolateral flattening, notching of posterior medial right lobe and parenchymal nodules, with or without manifestations of portal hypertension (portal-systemic collaterals, splenomegaly and/or ascites). (1,2)	Multiple sequences
Diffuse iron overload (present vs. absent)	Diffuse signal intensity drops of the liver parenchyma on in-phase images compared with the opposed-phase images. (1,2)	In-/opposed phase; T2WI
Diffuse fatty change (present vs. absent)	Diffuse signal intensity drops of the liver parenchyma on opposed-phase images compared with the in-phase images. (1,2)	In-/opposed phase; fat-suppressed images compared to non-fat-suppressed images with similar or identical weighting
Secondary manifestations of liver disease		
Ascites (present vs. absent)	Presence of free fluid in abdomen or pelvis. (19)	T2WI
Splenomegaly (present vs. absent)	Craniocaudal diameter of the spleen>12cm. (19,21,22)	Multiple sequences
Porto-systemic shunts (present vs. absent)	Enhancing tortuous channels in esophageal, epigastric, perisplenic, paraumbilical or retroperitoneal locations. (19)	Portal venous or delayed phase
Esophageal gastric varices (present vs. absent)	Discrete enhancing tortuous channel abutting the luminal surface of the esophageal or gastric wall or contacting/ protruding into luminal space. (19)	Portal venous or delayed phase

References:

1. CT/MRI Liver Imaging Reporting and Data System version 2018. American College of Radiology Web site. https://www.acr.org/Clinical-Resources/Reporting-andData- Systems/LI-RADS/CTMRI-LI-RADS-v2018. Accessed 1 September 2021.
2. LI-RADS Lexicon (terms and definitions). American College of Radiology Web site. https://www.acr.org/-/media/ACR/Files/RADS/LI-RADS/LIRADS-Lexicon-Table.pdf. Accessed 1 September 2021.
3. Fan Z, Jin M, Zhang L, et al. From clinical variables to multiomics analysis: a margin morphology-based gross classification system for hepatocellular carcinoma stratification. Gut. 2023 Nov;72(11):2149-2163.
4. Jiang H, Wei J, Fu F, et al. Predicting microvascular invasion in hepatocellular carcinoma: a dual-institution study on gadoxetate disodium-enhanced MRI. Liver Int. 2022;42(5):1158-1172.
5. Jiang H, Wei H, Yang T, et al. VICT2 Trait: Prognostic Alternative to Peritumoral Hepatobiliary Phase Hypointensity in HCC. Radiology. 2023 Feb 14:221835.
6. Rhee H, Cho ES, Nahm JH, et al. Gadoxetic acid-enhanced MRI of macrotrabecular-massive hepatocellular carcinoma and its prognostic implications. J Hepatol. 2021;74(1):109-121.
7. Segal E, Sirlin CB, Ooi C, Adler AS, Gollub J, Chen X, et al. Decoding global gene expression programs in liver cancer by noninvasive imaging. Nat Biotechnol 2007; 25:675–680.
8. Renzulli M, Brocchi S, Cucchetti A, Mazzotti F, Mosconi C Sportoletti C, et al. Can Current Preoperative Imaging Be Used to Detect Microvascular Invasion of Hepatocellular Carcinoma? Radiology 2016;279(2):432-442.
9. Lei Z, Li J, Wu D, Xia Y, Wang Q, Si A, et al. Nomogram for Preoperative Estimation of Microvascular Invasion Risk in Hepatitis B Virus-Related Hepatocellular Carcinoma Within the Milan Criteria. JAMA 2016;151(4):356-363.
10. Lee S, Kim SH, Lee JE, Sinn DH, Preoperative gadoxetic acid-enhanced MRI for predicting microvascular invasion in patients with single hepatocellular carcinoma. J Hepatol 2017;67(3):526-534.
11. An C, Kim DW, Park YN, Chung YE, Rhee H, Kim MJ. Single hepatocellular carcinoma: preoperative MR imaging to predict early recurrence after curative resection. Radiology 2015; 276:433–443.
12. Ronot M, Chernyak V, Burgoyne A, et al. Imaging to predict prognosis in hepatocellular carcinoma: current and future perspectives. Radiology. 2023;307(3):e221429.
13. Lee S, Kim SH, Lee JE, Sinn DH, Preoperative gadoxetic acid-enhanced MRI for predicting microvascular invasion in patients with single hepatocellular

carcinoma. J Hepatol 2017;67(3):526-534.

14. Adachi M, Kai K, Yamaji K, et al. Transferrin receptor 1 overexpression is associated with tumour de-differentiation and acts as a potential prognostic indicator of hepatocellular carcinoma. Histopathology. 2019;75(1):63-73.
15. Burt AD, Alves V, Bedossa P, et al. Data set for the reporting of intrahepatic cholangiocarcinoma, perihilar cholangiocarcinoma and hepatocellular carcinoma: recommendations from the International Collaboration on Cancer Reporting (ICCR). Histopathology. 2018; 73:369–85.
16. Marasco G, Colecchia A, Colli A, et al. Role of liver and spleen stiffness in predicting the recurrence of hepatocellular carcinoma after resection. J Hepatol. 2019;70(3):440-448.

Supplemental Table 3. Univariable Cox regression analyses for early recurrence-free survival based on the training dataset.

Variable	Univariate analyses		
	β	Hazard Ratio	*P* value
Clinical variables			
Age (y)	-0.0309	0.9695 (0.948-0.9915)	0.01
Age (> 55 vs. ≤55)	-0.7377	0.4782 (0.2818-0.8116)	0.01
Sex (male vs. female)	-0.2807	0.7553 (0.3732-1.5285)	0.44
The Barcelona Clinic Liver Cancer stage (A vs. 0)	0.7413	2.0987 (1.0685-4.1221）	0.03
Preoperative laboratory variables			
Alpha-fetoprotein (> 20 ng/mL vs. ≤ 20 ng/mL)	0.7285	2.0721 (1.2362-3.473)	0.01
Alpha-fetoprotein (>100 ng/mL vs. ≤100 ng/mL)	0.6435	1.9032 (1.1651-3.1088)	0.01
Alpha-fetoprotein (>400 ng/mL vs. ≤400 ng/mL)	0.822	2.2751 (1.3646-3.793）	0.002
Alanine aminotransferase (IU/L)	0.0019	1.0019 (0.9942-1.0097）	0.63
Alanine aminotransferase (>40 IU/L vs. ≤40 IU/L)	-0.1152	0.8912 (0.5252-1.5122）	0.67
Aspartate aminotransferase (IU/L)	0.0061	1.0061 (0.9996-1.0125）	0.06
Alanine aminotransferase (>35 IU/L vs. ≤35 IU/L)	0.4251	1.5297 (0.935-2.5028）	0.09
Albumin (g/L)	0.0246	1.0249 (0.9769-1.0753）	0.32
Albumin (>40 g/L vs. ≤40g/L)	0.0342	1.0348 (0.5628-1.9029）	0.91
Total bilirubin (umol/L)	-0.0326	0.9679 (0.9266-1.0111）	0.14
Total bilirubin (>21 umol/L vs. ≤21 umol/L)	-0.7888	0.4544 (0.196-1.0536）	0.07
Total bilirubin (>28 umol/L vs. ≤28 umol/L)	0.0329	1.0335 (0.2526-4.229）	0.96
The albumin-bilirubin score	-0.4023	0.6688 (0.3788-1.1808）	0.17
The albumin-bilirubin grade (2, 3 vs. 1)	0.3268	1.3866 (0.7541-2.5497）	0.29
Platelet count (^9/L)	0.0005	1.0005 (0.9959-1.0051）	0.84
Platelet count (>120^9/L vs. ≤120^9/L)	-0.1655	0.8474 (0.5186-1.3849）	0.51
Platelet count (>135^9/L vs. ≤135^9/L)	-0.1293	0.8787 (0.5317-1.4521）	0.61
Platelet count (>150^9/L vs. ≤150^9/L)	-0.0344	0.9662 (0.5651-1.6519）	0.90
Platelet count (>300^9/L vs. ≤300^9/L)	1.3198	3.7428 (0.5133-27.2892）	0.19
Prothrombin time (s)	0.0183	1.0185 (0.8139-1.2745）	0.87
Prothrombin time (>12.8 s vs. ≤12.8 s)	-0.1555	0.8559 (0.4358-1.6811）	0.65
HBV-DNA (>10000 IU/ml vs. ≤ 10000 IU/ml)	0.2008	1.2223 (0.6521-2.2911）	0.53
PIVKA-II (>40 mAU/ml vs. ≤ 40 mAU/ml)	1.0485	2.8533 (1.3597-5.9875）	0.01
PIVKA-II (>200 mAU/ml vs. ≤ 200 mAU/ml)	1.0155	2.7608(1.6677-4.5704)	<0.001
Preoperative imaging features			
Tumor location^			
Left lateral section	*Ref*	*Ref*	...
Caudate lobe	5.2665	193.735 (10.9194-3437.3151）	<0.001
Medial section	-0.6197	0.5381 (0.2476-1.1692）	0.12
Right posterior section	-0.435	0.6472 (0.2963-1.414）	0.28
Cross-regional involvement	0.1469	1.1582 (0.4797-2.7963）	0.74
Imaging features related to tumor aggressiveness			
LI-RADS major features			
Nonrim arterial phase hyperenhancement (present vs. absent)	-0.3126	0.7315 (0.2934-1.8242）	0.50
Nonperipheral washout (present vs. absent)	0.773	2.1663 (1.0324-4.5454）	0.04
Enhancing capsule (present vs. absent)	-0.3867	0.6793 (0.3237-1.4257）	0.31
Tumor size (> 3 cm vs. ≤ 3 cm)	0.6998	2.0134 (1.1945-3.3937）	0.01
LI-RADS ancillary features			
Diffusion restriction (present vs. absent)	...	...	...
Mild-moderate T2 hyperintensity (present vs. absent)	0.6369	1.8907 (0.2621-13.6388）	0.53
Marked T2 hyperintensity (present vs. absent)	...	...	...
Corona enhancement (present vs. absent)	-0.0069	0.9931 (0.57-1.7305）	0.98
Nonenhancing capsule (present vs. absent)	1.1118	3.0397 (0.415-22.2666）	0.27
Nodule-in-nodule architecture (present vs. absent)	0.2993	1.3489 (0.7742-2.35）	0.29
Mosaic architecture (present vs. absent)	0.835	2.3048 (1.3887-3.8253）	0.001
Blood products in mass (present vs. absent)	0.6338	1.8848 (1.1471-3.0968）	0.01
Fat in mass, more than adjacent liver (present vs. absent)	0.4683	1.5972 (0.966-2.6409）	0.07
Fat sparing in solid mass (present vs. absent)	0.0561	1.0578 (0.3844-2.9109）	0.91
Iron sparing in solid mass (present vs. absent)	0.2365	1.2668 (0.6761-2.3736）	0.46
TP hypointensity (preset vs. absent) †	-0.0729	0.9295 (0.1261-6.8536)	0.94

HBP hypointensity (preset vs. absent) †	...	...	...
Iron in mass, more than liver (present vs. absent)	...	...	...
LR-M features			
Rim arterial phase hyperenhancement (present vs. absent)	0.5643	1.7582 (0.7051-4.3843）	0.23
Peripheral washout (present vs. absent)	1.0261	2.7901 (0.8736-8.9117）	0.08
Delayed central enhancement (present vs. absent)	...	...	...
Targetoid restriction (present vs. absent)	0.8756	2.4004 (0.5863-9.828）	0.22
Targetoid TP or HBP appearance (present vs. absent) †	1.6196	5.0421 (0.6628-38.3533)	0.12
Marked diffusion restriction (present vs. absent)	0.4926	1.6366 (0.9164-2.9226）	0.10
Infiltrative appearance (present vs. absent)	1.5491	4.7073 (2.009-11.0301）	<0.001
Necrosis or severe ischemia (present vs. absent)	0.639	1.8947 (1.1578-3.1006）	0.01
Other previously-reported prognostic features of the tumor			
Pre-contrast T1-weighted hypo intensity (present vs. absent)	1.1692	3.2193 (0.4462-23.2284）	0.25
Pre-contrast T1-weighted hyper intensity (present vs. absent)	1.7248	5.6112 (0.5084-61.9315）	0.16
T2-weighted peritumoral hyperintensity (present vs. absent)	0.5117	1.668 (0.9664-2.8791）	0.07
Portal venous phase peritumoral hypoenhancement (present vs. absent)	0.9781	2.6593 (1.5835-4.4659）	<0.001
Arterial phase hyperenhancement (<50% vs. ≥50%)	-0.2841	0.7527 (0.4536-1.2491	0.27
Intratumoral artery (present vs. absent)	1.0163	2.763 (1.6907-4.5155）	<0.001
Complete capsule (present vs. absent)	-0.4968	0.6085 (0.3098-1.1952）	0.15
Non-smooth tumor margin (present vs. absent)	0.6658	1.9461 (0.9905-3.8236）	0.05
Bilobar involvement (present vs. absent)	0.6322	1.8818 (0.6835-5.1809）	0.22
Radiological involvement of liver capsule (present vs. absent)	0.8533	2.3473 (1.3328-4.1342）	0.003
Markedly low ADC value (present vs. absent)	0.7184	2.0511 (0.935-4.4995）	0.07
HBP peritumoral hypointensity (present vs. absent) †	0.4789	1.6143 (0.7469 - 3.4894)	0.22
Early peritumoral hyperenhancement (present vs. absent)	0.1724	1.1881 (0.4903-2.8791）	0.70
Single nodular growth type (present vs. absent)	0.4255	1.5304 (0.9333-2.5094）	0.09
Tumor growth subtype			
Single nodular type	*Ref*	*Ref*	
Single nodule type with extranodular growth	0.3655	1.4413 (0.8705-2.3862）	0.16
Confluent multi-nodular or infiltrative type	1.8994	6.6822 (2.0201-22.1039）	0.002
Imaging features related to underlying liver condition			
The background liver-related features			
Radiologically-evident cirrhosis (present vs. absent)	0.0834	1.087 (0.6303-1.8745）	0.76
Diffuse fatty change (present vs. absent)	0.2661	1.3048 (0.6445-2.6416）	0.46
Diffuse iron overload (present vs. absent)	-0.486	0.6151 (0.2805-1.3486）	0.23
Secondary manifestations of liver disease			
Ascites (present vs. absent)	-0.2926	0.7463 (0.2341-2.3792）	0.62
Splenomegaly (present vs. absent)	-0.1155	0.8909 (0.5389-1.4727）	0.65
Porto-systemic shunts (present vs. absent)	0.0182	1.0184 (0.6138-1.6897）	0.94
Esophageal gastric varices (present vs. absent)	0.2556	1.2913 (0.7862-2.121）	0.31

Note. —Unless stated otherwise, data in parentheses are 95% confidence intervals.

†Data are presented for training dataset patients who underwent hepatobiliary contrast agent-enhanced MRI (N=79)

^Caudate lobe refers to segments 1, left lateral section refers to segments 2,3, medial section refers to segments 4,5 and 8, and right posterior section refers to segments 6 and 7. Cross-regional involvement implies the involvement of more than one of the aforementioned locations.

HR = hazard ratio, Ref = reference.

Supplemental Table 4. Frequencies and interrater agreement for all analyzed MRI features.

Features	frequency	K	agreement
Tumor location			
Caudate lobe	3 (0.7)		
Left lateral section	64 (15.2)		
Medial section	176 (41.9)	0.891 (0.873-0.912)	Excellent
Right posterior section	134 (31.9)		
Cross-regional involvement	43 (10.2)		
Imaging features related to tumor aggressiveness			
LI-RADS major features			
Nonrim arterial phase hyperenhancement (present vs. absent)	392 (93.6)	0.441 (0.386-0.497)	Moderate
Nonperipheral washout (present vs. absent)	321 (76.6)	0.506 (0.451-0.561)	Moderate
Enhancing capsule (present vs. absent)	372 (88.8)	0.420 (0.365-0.475)	Moderate
Tumor size (< 3 cm vs. ³ 3 cm)	213 (50.8)	0.905(0.903-0.907)	Excellent
LI-RADS ancillary features			
Diffusion restriction (present vs. absent)	419 (100)	0.986 (0.982-0.993)	Excellent
Mild-moderate T2 hyperintensity (present vs. absent)	405 (96.7)	0.504 (0.448-0.559)	Moderate
Marked T2 hyperintensity (present vs. absent)	9 (2.1)	0.457 (0.401-0.512)	Moderate
Corona enhancement (present vs. absent)	116 (27.7)	0.333 (0.278-0.388)	Fair
Nonenhancing capsule (present vs. absent)	11 (2.6)	0.418 (0.363-0.474)	Moderate
Nodule-in-nodule architecture (present vs. absent)	98 (23.4)	0.41 (0.355-0.465)	Moderate
Mosaic architecture (present vs. absent)	90 (21.5)	0.497 (0.441-0.552)	Moderate
Blood products in mass (present vs. absent)	101 (24.1)	0.733 (0.678-0.788)	Substantial
Fat in mass, more than adjacent liver (present vs. absent)	134 (32.0)	0.471 (0.416-0.526)	Moderate
Fat sparing in solid mass (present vs. absent)	19 (4.5)	0.121 (0.066-0.177)	Slight
Iron sparing in solid mass (present vs. absent)	72 (17.2)	0.175 (0.12-0.23)	Slight
TP hypointensity (preset vs. absent) [†]	157 (37.5)	0.872 (0.817-0.927)	Excellent
HBP hypointensity (preset vs. absent) [†]	165 (39.4)	0.825 (0.8-0.86)	Excellent
Iron in mass, more than liver (present vs. absent)	2 (0.5)	0.08 (0.025-0.135)	Slight
Parallels blood pool enhancement (present vs. absent)	0 (0)	0.332 (0.283-0.381)	Fair
Undistorted vessels (present vs. absent)	0 (0)	0.272 (0.223-0.321)	Fair
LR-M features			
Rim arterial phase hyperenhancement (present vs. absent)	21 (5.0)	0.447 (0.391-0.502)	Moderate
Peripheral washout (present vs. absent)	5 (1.2)	0.955 (0.936-0.972)	Excellent
Delayed central enhancement (present vs. absent)	5 (1.2)	0.265 (0.21-0.321)	Fair
Targetoid restriction (present vs. absent)	4 (1.0)	0.37 (0.315-0.426)	Fair
Targetoid TP or HBP appearance (present vs. absent) [†]	3 (0.7)	0.776 (0.721-0.831)	Substantial
Marked diffusion restriction (present vs. absent)	76 (18.1)	0.704 (0.658-0.734)	Substantial
Infiltrative appearance (present vs. absent)	15 (3.6)	0.497 (0.442-0.552)	Moderate
Necrosis or severe ischemia (present vs. absent)	119 (28.4)	0.699 (0.644-0.754)	Substantial
Other tumor-related prognostic features			
Pre-contrast T1-weighted hypo intensity	398 (95.0)	0.886 (0.857-0.919)	Excellent
T2-weighted peritumoral hyperintensity (present vs. absent)	68 (16.2)	0.497 (0.442-0.552)	Moderate
Portal venous phase peritumoral hypoenhancement (present vs. absent)	69 (16.5)	0.521 (0.466-0.577)	Moderate
Arterial phase hyperenhancement (<50% vs. ³ 50%)	305 (72.8)	0.775 (0.747-0.806)	Substantial
Intratumoral artery (present vs. absent)	119 (28.4)	0.598 (0.542-0.653)	Moderate
Complete capsule (present vs. absent)	113 (27.0)	0.393 (0.338-0.449)	Fair
Non-smooth tumor margin (present vs. absent)	304 (72.6)	0.499 (0.444-0.554)	Moderate
Bilobar involvement	14 (3.3)	0.943 (0.925-0.962)	Excellent
Radiogical involvement of liver capsule	265 (63.2)	0.623 (0.568-0.678)	Substantial
markedly low ADC value (present vs. absent)	28 (6.7)	0.737 (0.71-0.765)	Substantial
HBP peritumoral hypointensity (present vs. absent) [†]	56 (13.4)	0.74 (0.685-0.796)	Substantial
Early peritumoral hyperenhancement (present vs. absent)	32 (7.6)	0.405 (0.336-0.465)	Moderate
Tumor growth subtype			
Single nodular type	225 (53.7)		
Single nodule type with extranodular growth	186 (44.4)	0.646 (0.627-0.684)	Substantial
Confluent multi-nodular or infiltrative type	8 (1.9)		
Imaging features related with the underlying liver condition			

The background liver-related features			
Radiologically-evident cirrhosis (present vs. absent)	301 (71.8)	0.445 (0.39-0.5)	Moderate
Diffuse iron overload (present vs. absent)	67 (16.0)	0.436 (0.381-0.491)	Moderate
Diffuse fatty change (present vs. absent)	37 (8.8)	0.568 (0.512-0.623)	Moderate
Secondary manifestations of liver disease			
Ascites (present vs. absent)	19 (4.5)	0.346 (0.291-0.401)	Fair
Splenomegaly (present vs. absent)	200 (47.7)	0.705 (0.669-0.721)	Substantial
Porto-systemic shunts (present vs. absent)	234 (55.8)	0.33 (0.275-0.385)	Fair
Esophageal gastric varices (present vs. absent)	152 (36.3)	0.343 (0.288-0.398)	Fair

Note. —Unless stated otherwise, data in parentheses are percentages or 95% confidence intervals. R1, R2, and R3 were reviewers with 7, 10, and 3 years of experiences in liver MRI, respectively.

*Interobserver agreement was investigated by computing the Fleiss'κ value for binary variables, the intraclass correlation coefficient (ICC) for continuous variables, and the weighted κ value for ordinal/categorical variables. Agreement was considered slight (κ or ICC < 0.2), fair (κ or ICC: 0.2-0.4), moderate (κ or ICC: 0.4-0.6), substantial (κ or ICC: 0.6-0.8), or excellent (κ or ICC > 0.8).

†Analyses were conducted for patients who underwent hepatobiliary contrast agent-enhanced MRI (n=79).

LI-RADS = Liver Imaging Reporting and Data System; TP = transitional phase; HBP = hepatobiliary phase.

Supplement Table 5. The pathological characteristics between different *MARGIN* risk groups for patients with narrow resection margins.

Characteristic	Low-risk Group (n=220)	High-risk Group (n=62)	P-value
Microvascular invasion, Present$	52 (24.8)	38 (61.3)	**<0.001**
Tumor differentiation, grade 3-4$	60 (27.3)	28(45.2)	**0.01**
Liver capsular involvement, Positive$	68 (31.1)	37 (59.7)	**<0.001**
Satellite nodules, Positive$	6 (2.8)	5 (8.6)	0.06

Categorical variables are the number of patients, with percentages in parentheses.

P values were computed by comparing date between the low-risk and high-risk groups.

$Date were presented for patients who had complete documentation.

Supplemental Table 6. Characteristics of the propensity score-matched cohort.

Characteristic	Matched Narrow Margin Group (n=137)	Wide Margin Group (n=137)	P-value*
Patient demographics			
Age, years	54 (45-62)	54 (47-62)	0.61
Sex, male	116 (84.7)	117 (85.4)	0.87
Chronic hepatitis B, Positive	112 (81.8)	108 (78.8)	0.54
Pathological cirrhosis, Positive	77 (59.2)	78 (59.5)	0.96
Laboratory parameters			
ALT, IU/L	31 (21-43.5)	32 (23-45)	0.81
AST, IU/L	29 (23-40)	28 (22.5-35.5)	0.33
Albumin, g/dL	45.2 (41.35-48)	44.3 (42.05-46.75)	0.30
Total bilirubin, μmol/L	14 (10.3-20.1)	13.1 (9.6-17.4)	0.19
Prothrombin time, s	11.6 (11.1-12.5)	11.4 (10.9-12.2)	0.16
Tumor characteristics			
Poorly-differentiated, Positive$	44 (32.4)	37 (27.2)	0.35
Microvasular invasion, Present$	39 (35.1)	42 (35.9)	0.90
Liver capsular involvement, Positive$	46 (34.8)	59 (44.0)	0.13
Satellite nodules, Positive$	5 (4.0)	6 (4.8)	0.76
Perioperative data			
Resection extent, Major resection$	12 (9.2)	21 (15.7)	0.11
Type of operation, Minimally invasive$	63 (46.0)	71 (51.8)	0.33
Operative blood loss, mL$	100 (50-300)	100 (50-200)	0.42
Perioperative blood transfusion, yes$	6 (4.5)	2 (1.5)	0.17
Operative time, >3 hours	47 (35.6)	47 (35.3)	0.96
Hospitalization time, days	7 (7-10)	7 (7-9)	0.19
Postoperative adjuvant therapy, yes$	25 (18.2)	29(21.2)	0.54
Clinical outcome			
Follow-up time, months	31.7 (17-46.05)	29.5 (17.58-44.50)	0.67
Early recurrence location			0.10
Without early recurrence	100 (73.0)	112 (81.8)	
Intrahepatic recurrence	24 (17.5)	18 (13.1)	
Macrovascular invasion	2 (1.5)	0 (0.0)	
Extrahepatic metastases	3 (2.2)	5 (3.6)	
Multiple metastases	8 (5.8)	2 (1.5)	

Note. —Unless stated otherwise, data in parentheses are interquartile range, percentages, or 95% confidence intervals.

*Differences were compared with the paired t test or Wilcoxon signed-rank test for continuous variables, and with the McNemar test for categorical variables, as appropriate.

$Date were presented for patients who had complete documentation.

Supplemental Table 7. Univariable Cox regression analyses for overall recurrence-free survival in *MARGIN*-predicted high-risk (MARGIN score ≥-1.3 points) patients.

**Variable**	**Univariate analyses**	
	**Hazard Ratio**	***P* value**
Preoperative clinical and laboratory variables		
Sex, male*	0.52 (0.18-1.56)	0.24
Platelet count (^9/L)	1.00 (1.00-1.01)	0.24
Prothrombin time, s	1.16 (0.80-1.67)	0.43
AST, IU/L	1.00 (0.99-1.02)	0.66
ALT, IU/L	1.00 (0.98-1.01)	0.81
AST/ALT	2.10 (0.45-9.74)	0.34
Albumin, g/dL	1.03 (0.96-1.10)	0.38
Total bilirubin (umol/L)	0.99 (0.93-1.05)	0.66
Child-Pugh class (B vs. A)	3.69 (0.82-16.57)	0.09
ALBI score	0.66 (0.30-1.45)	0.30
ERASL-pre score	1.48 (0.68-3.24)	0.33
Tumor size, cm	1.06 (0.95-1.18)	0.27
Tumor size ≤ 2 cm	*Ref*	*Ref*
Tumor size 2-5 cm	0.53 (0.06-4.33)	0.55
Tumor size > 5 cm	1.26 (0.16-9.99)	0.83
The Barcelona Clinic Liver Cancer stage (A vs. 0)	0.81 (0.11-6.22)	0.84
Pathological variables		
Pathological liver cirrhosis, Positive$	1.15 (0.51-2.62)	0.74
Pathological liver capsular involvement, Positive$	1.07 (0.44-2.60)	0.89
Microvascular invasion, Present$	1.78 (0.76-4.13)	0.18
Tumor differentiation, Grade 3-4$	1.12 (0.49-2.56)	0.79
Satellite nodules, Present$	2.83 (0.65-12.24)	0.16
Treatment options		
Margin width, wide-margin	0.29 (0.12-0.72)	**0.007**
Resection extent, major resection$	1.05 (0.42-2.62)	0.92
Type of operation, minimally invasive	0.73 (0.32-1.70)	0.47
Postoperative adjuvant therapy, yes$	1.32 (0.43-4.03)	0.63

Note. —Unless stated otherwise, data in parentheses are 95% confidence intervals.

$Date were presented for patients who had complete documentation.

HR = hazard ratio, Ref = reference.

Supplementary Figure 1. Kaplan-Meier curves for early (≤2 years) recurrence-free survival (A) and overall recurrence-free survival (B) for patients with narrow resection margins and wide resection margins.

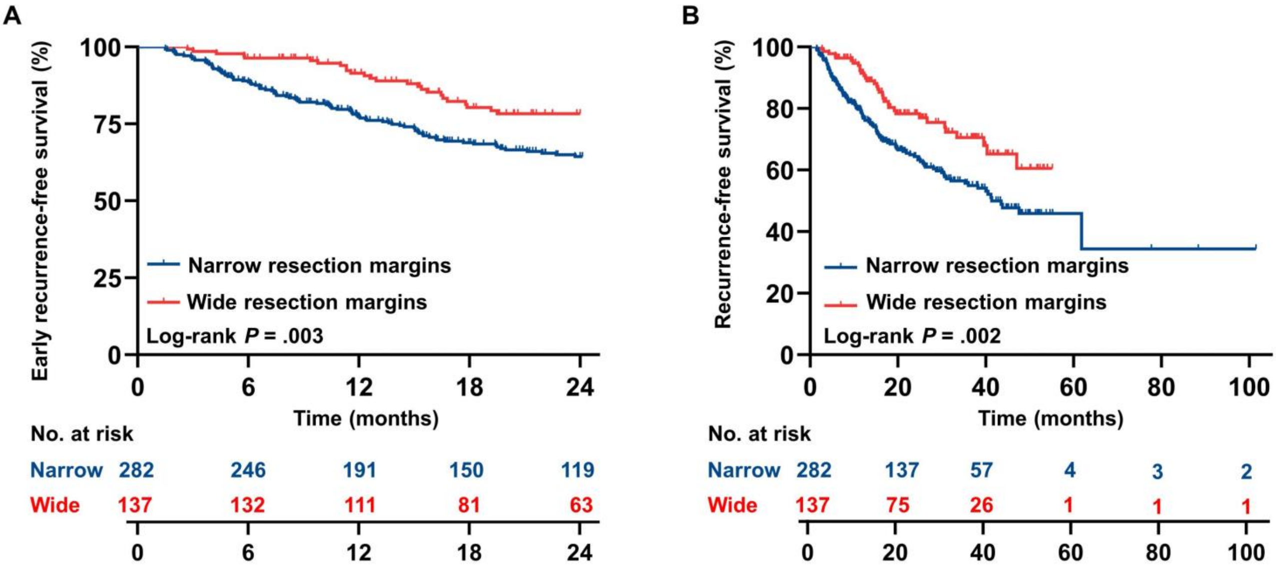

Supplementary Figure 2. Performance of the *MARGIN* score in predicting 2-year RFS in the training and testing datasets. The calibration curve (A, B) revealed good conformity between the predicted and observed outcomes. The MARGIN score had a higher net benefit than “treat all” or “treat none” strategies across the majority range of reasonable threshold probabilities (C, D).

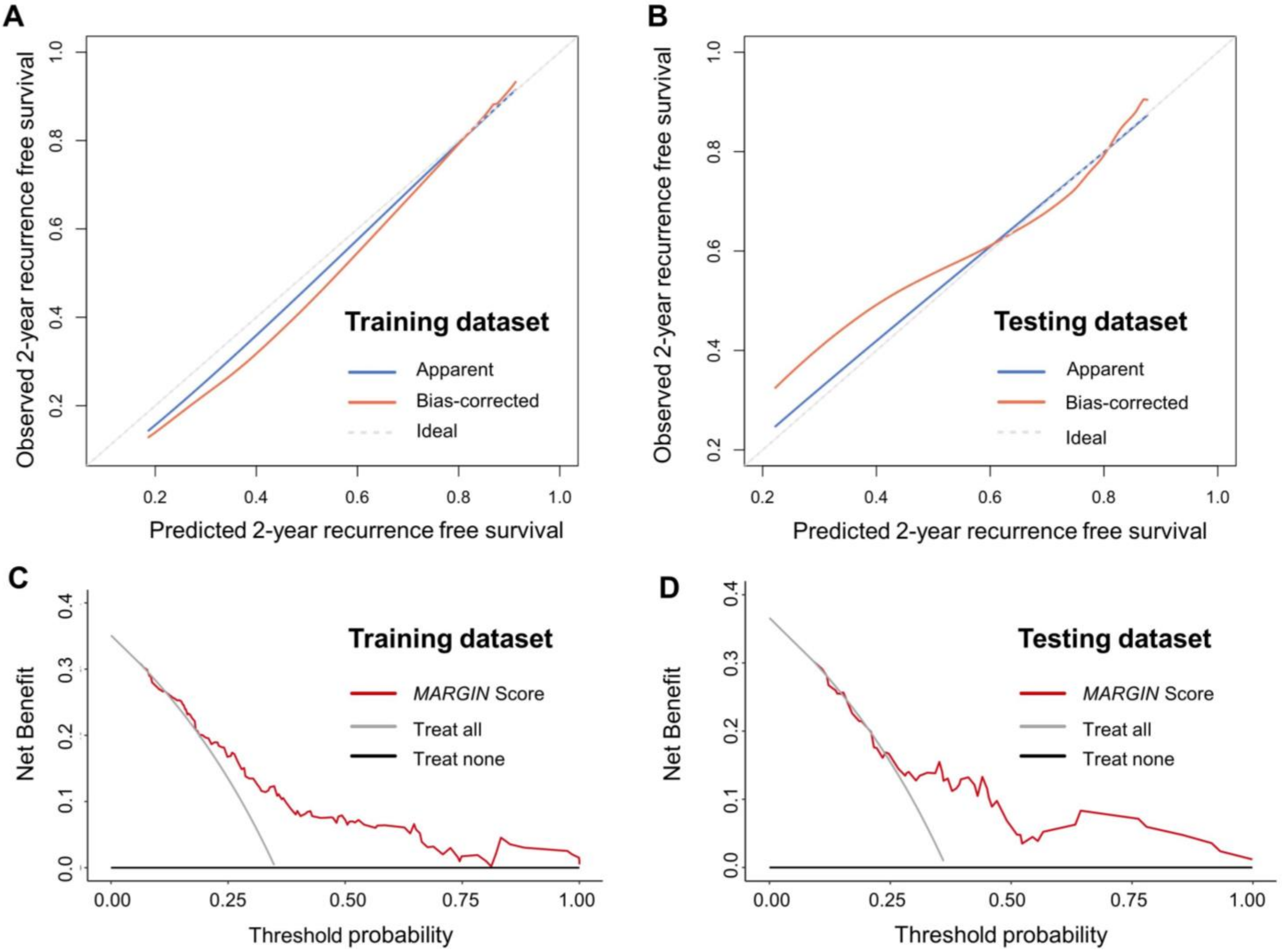

Supplemental Figure 3. Early (≤2 years) recurrence-free survival outcomes for the ERASL-predicted low-risk patients. Survival outcomes are plotted as Kaplan-Meier curves for MARGIN high-risk (A) and MARGIN low-risk (B) groups.

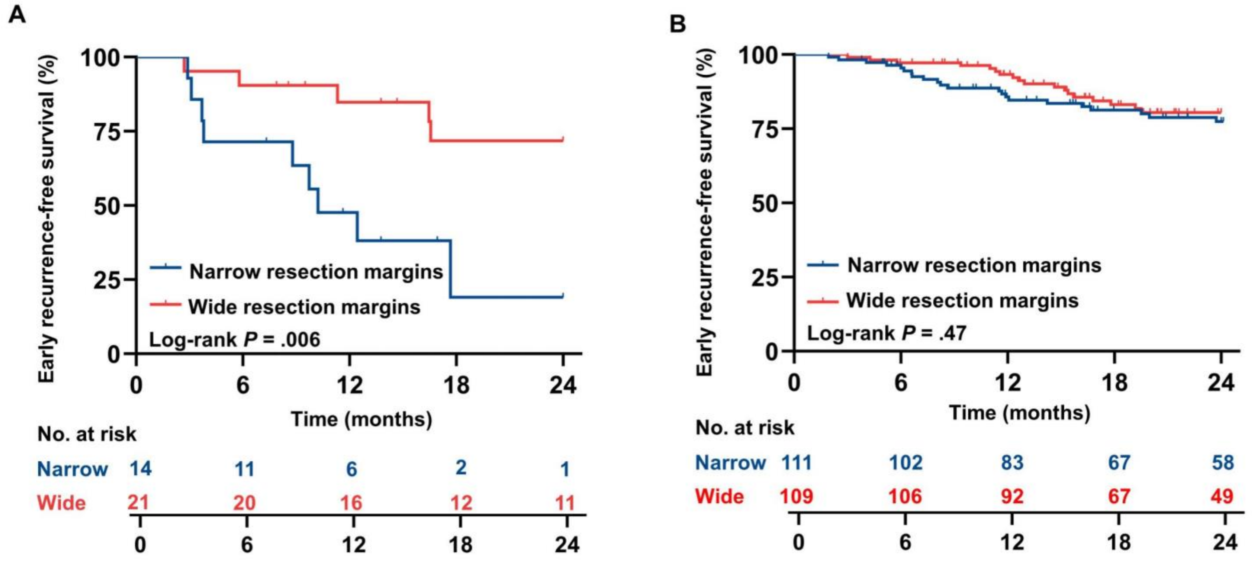

Supplement: Supplementary file 1 — ELECTRONIC SUPPLEMENTARY MATERIAL [file 330_2024_11043_MOESM1_ESM.pdf]
